# Prediction of Potential Habitat Distribution of *Cibotium barometz* (L.) J. Sm. Under Climate Change Based on a Multi-Model Ensemble Framework

**DOI:** 10.3390/biology15090692

**Published:** 2026-04-28

**Authors:** Heng Jiang, Yunfang Zhang, Tao Li, Shuang Zhang, Ying Liu, Yvdan Chen, Minjing Deng, Kunhua Wei, Quan Yang

**Affiliations:** 1 Key Laboratory of State Administration of Traditional Chinese Medicine for Production & Development of Cantonese Medicinal Materials, Guangzhou Comprehensive Experimental Station of National Industrial Technology System for Chinese Materia Medica, Guangdong Engineering Research Center of Good Agricultural Practice & Comprehensive Development for Cantonese Medicinal Materials, School of Chinese Materia Medica, Guangdong Pharmaceutical University, Guangzhou 510006, China; jh15874556538@163.com (H.J.); 16696379526@163.com (Y.Z.); 15362935395@163.com (T.L.); wenliang57321@163.com (S.Z.); 19808032056@163.com (Y.L.); 18934114933@163.com (Y.C.); m19865200250@163.com (M.D.); 2 Yunfu Germplasm Resource Management Center, Yunfu 527300, China

**Keywords:** multi-model ensemble system, ArcGIS, *Cibotium barometz* (L.) J. Sm., MaxEnt optimization, potential geographic distribution

## Abstract

Understanding how medicinal plant distributions shift in response to climate change is essential for developing forward-looking conservation strategies. *Cibotium barometz* (L.) J. Sm., a tree fern from the family Dicksoniaceae, is not only ecologically significant but also holds considerable medicinal value. Despite its importance, wild populations of this species have been steadily declining due to ongoing habitat loss and unsustainable harvesting. To address this concern, we constructed a multi-model ensemble framework that integrated nine different algorithms, including Generalized Linear Models, various machine learning approaches, and a MaxEnt model optimized through ENMeval using a regularization multiplier of 2 and a feature class of LQH. Using this modeling framework, we simulated the habitat suitability dynamics of *C. barometz* under current climate conditions (1970–2000) and two future periods (2050s and 2090s) across four Shared Socioeconomic Pathways (SSP126, SSP245, SSP370, and SSP585). Our analysis identified water availability and low temperature stress as the primary factors limiting the species’ distribution. The suitable range for precipitation during the driest quarter extends from 3.25 to 640.20 mm, with optimal conditions occurring when precipitation reaches at least 96.84 mm. Annual precipitation suitable for the species lies between 74.58 and 4209.60 mm, and the most favorable range falls between 3834.10 and 4209.60 mm. While the minimum temperature of the coldest month can vary from −35.41 to 22.35 °C, optimal survival requires temperatures of 8.79 °C or higher. In addition, the species grows best within an annual temperature range of 16.25 to 27.92 °C, with an optimum around 20.47 °C. Projections based on the multi model ensemble suggest that future climate warming may lead to a southwestward shift in the centroid of suitable habitat for this species. By the 2090s, under the SSP245, SSP370, and SSP585 scenarios, the centroid shifts southwestward by 331.3 km, 335.1 km, and 180.2 km, respectively. Meanwhile, areas with high habitat suitability are expected to retreat toward mid-to-high elevation zones, especially in southeastern Yunnan, southern Guizhou, and western Guangxi. The effects of different emission pathways vary considerably; under the high-emission SSP585 scenario, the reduction in total suitable area is projected to be more severe and habitat fragmentation more extensive compared to the low-emission SSP126 pathway. In contrast, implementing ambitious emissions reduction measures could play a key role in supporting the long-term stability of *C. barometz* populations. This study clarifies how this species responds to climate change and the spatial strategies it may adopt, providing a scientific basis and spatial references for conserving its germplasm resources, restoring its habitats, and advancing its sustainable use.

## Simple Summary

Understanding how climate change reshapes the distribution of *Cibotium barometz* (L.) J. Sm., a medicinal fern with both therapeutic and ecological roles, is essential for its future conservation. This study employed an integrated multi-model approach to project the species’ habitat shifts under various climate scenarios. Our findings indicate a pronounced southwestward migration of suitable areas towards higher altitudes in Yunnan, Guizhou, and Guangxi, with habitat loss and fragmentation intensifying under higher emission pathways. These results provide crucial spatial insights for guiding the long-term protection and sustainable management of this valuable species.

## 1. Introduction

As a representative species of traditional medicinal ferns in China, *Cibotium barometz* (L.) J. Sm. is a perennial plant belonging to the genus *Cibotium* (family Dicksoniaceae). The genus *Cibotium* comprises about 20 species distributed from Southeast Asia to Oceania and Central America; in China, only *C. barometz* occurs, and it is officially designated as a Class II National Protected Plant [1]. According to the *Flora of China*, this species is naturally distributed across several southern provinces, including Fujian, Taiwan, Guangdong, Guangxi, Yunnan, and Guizhou, where it typically grows in shaded valleys or sparse woodlands near streams [2]. As a typical shade-tolerant fern, *C. barometz* is highly dependent on warm and humid environments. The earliest written record of *C. barometz* can be traced back to the *Shennong’s Herbal Classic*, where it was categorized as a medium-grade medicinal substance. The common name “dog spine” originates from the distinctive appearance of its rhizome, which is thickly coated with golden-yellow hairs and visually resembles the backbone of a canine [3]. As a frequently utilized traditional Chinese medicinal material, *C. barometz* exhibits a broad spectrum of biological activities [4,5,6], and its golden trichomes are used for external hemostasis, while the rhizome also holds potential for horticultural ornamentation and ecological restoration [7,8].

The *Pharmacopoeia of the People’s Republic of China* documents the traditional applications of *C. barometz*, noting its role in nourishing the liver and kidneys, alleviating discomfort in the lower back and knees, and addressing wind-dampness conditions. Clinically, it is often recommended for symptoms including lumbar soreness, limb weakness, rheumatic pain, and bleeding caused by trauma [9]. Contemporary pharmacological studies have confirmed that *C. barometz* possesses a broad range of biological activities, including anti-inflammatory, immunomodulatory, and anti-osteoporotic effects [10,11,12]. Beyond its medicinal applications, *C. barometz* is endowed with notable economic and ecological functions. As an arborescent perennial fern, it is characterized by a robust rhizome, an aesthetically pleasing growth form, and golden trichomes, rendering it not only a medicinal resource but also a widely utilized ornamental plant and a material for handicrafts, thereby sustaining a relatively stable market demand. Despite this value, the resource supply for this species has long depended on wild collection, with no reports of large-scale artificial cultivation to date [13]. According to field surveys conducted across China, the natural distribution of *C. barometz* spans multiple southern provinces and regions, including Fujian, Guangxi, Guizhou, Yunnan, Guangdong, Jiangxi, Hunan, Sichuan, Chongqing, and Zhejiang. It is estimated that the total area occupied by this species amounts to roughly 7000 hectares, supporting an overall resource reserve exceeding 5 million kg. The current annual yield is approximately 850,000 kg, a figure that largely meets the average yearly market demand of 800,000 kg for medicinal purposes [13]. In recognition of its ecological and economic importance, *C. barometz* has been designated as a Class II National Key Protected Wild Plant in China [14], highlighting the urgent need for effective resource conservation and sustainable management.

However, due to a combination of factors, including uncontrolled harvesting, habitat destruction, and a relatively long natural regeneration cycle (approximately 5–7 years), wild resources of *C. barometz* have exhibited a persistent declining trend. A case in point is Zhanjiang in Guangdong Province, where the historical peak annual procurement reached 300,000 kg, whereas in recent years, this figure has drastically decreased to less than 100,000 kg [15]. Concurrently, market prices have shown a continuous upward trajectory: the procurement price per kg was 0.81 RMB in 1983, and by 1994, it had escalated to 3.30 RMB, representing more than a threefold increase over the decade [15]. The growing imbalance between market demand and available supply, together with sharp swings in price, underscores the real-world difficulties involved in protecting and sustainably using *C. barometz* resources. Addressing this situation calls for immediate action to secure a stable market supply and support the species’ long-term survival through evidence-based resource evaluation and thoughtful production strategies. It should be noted that the occurrence data used in this study were primarily derived from specimen records (CVH, NSII, GBIF), which may be affected by historical harvesting activities. In areas with historically high harvesting (e.g., Zhanjiang, Guangdong), severe population declines may have led to missing occurrence records, even though environmental conditions remain suitable. Such “anthropogenic false absences” can lead to underestimation of the species’ actual suitable distribution [16]. Therefore, our predictions may be conservative in areas with high anthropogenic pressure.

Over the past fifty years, climate change has led to an approximate 1.2 °C increase in the average annual temperature across China’s subtropical areas, while extreme precipitation events have also become more frequent [17,18]. As a typical shade-tolerant fern, *C. barometz* exhibits a pronounced dependence on stable temperature and humidity conditions for spore germination and gametophyte development. Previous studies have shown that temperature increases are associated with a contraction of suitable habitats toward higher elevations, and drought stress is associated with inhibition of sporophyte growth (a > 40% increase in the leaf wilting index) [19]. Furthermore, altered precipitation patterns have been associated with increased habitat fragmentation; for example, a 30% reduction in wild populations was observed in the Wuyi Mountain area of Fujian Province during consecutive drought years [20,21]. Changes in soil microbial community structure, such as a decrease in the abundance of arbuscular mycorrhizal fungi, further compromise its nutrient absorption capacity [22]. Moreover, anthropogenic disturbances (e.g., expansion of fast-growing plantations such as eucalyptus) and land-use change jointly affect species distribution; an approximate 50% reduction in the core distribution area of *C. barometz* has been observed in southern Yunnan [23]. It should be noted that these correlative observations cannot be attributed solely to a single climatic factor, as confounding factors such as land-use change also play important roles. Therefore, to preserve the genetic diversity and ensure the sustainable utilization of this species, it is imperative to establish climate-adaptive protected area networks and advance research on ex situ spore conservation and artificial propagation techniques [8,24].

In recent decades, the growing influence of global climate change is causing many rare and endangered species to experience rapid contraction or movement of their natural habitats; e.g., Panero et al. [25] found that the suitable habitat of the Mediterranean endemic *Primula palinuri* is projected to contract substantially under future climate scenarios; Dong et al. [26] predicted that the suitable distribution area of the rare and endangered *Fritillaria przewalskii* will shift towards higher elevations and latitudes. Ecological niche models have become important tools for assessing how species respond to environmental change. These models work by establishing links between where species occur and the surrounding environmental conditions, making it possible to evaluate shifts in habitat suitability, habitat quality, and climate driven responses [27,28,29]. Nevertheless, projections based on conventional single-model methods often carry substantial uncertainty, largely stemming from inherent differences among algorithms [30]. Therefore, this study adopted a multi-model ensemble strategy within the Biomod2 framework [31,32,33]. Nine algorithms were initially evaluated: GLM, GAM, GBM, CTA, ANN, SRE, MARS, RF, and MaxEnt. Among them, GLM and GAM are suitable for interpreting non-linear relationships; GBM, RF, and ANN excel at capturing complex interactions; CTA and MARS provide decision tree visualization and adaptive regression capabilities, respectively; SRE is a simple envelope-based model; MaxEnt performs robustly with small sample sizes [34,35]. Based on performance thresholds (AUC ≥ 0.80, TSS ≥ 0.60, Kappa ≥ 0.50), models were selected and combined using equal-weight averaging to generate consensus predictions, thereby reducing uncertainty associated with any single model [36].

With these considerations in mind, the present study seeks to accomplish several related goals. We first aim to pinpoint the key environmental variables that influence where *C. barometz* is found and to assess their relative importance. Building on this, we then use current climate data to simulate and map the potential range of this species across China. We will also examine how the suitable habitats of this species evolve over time by considering two future periods, the 2050s and 2090s, under a range of climate scenarios including SSP126, SSP245, SSP370, and SSP585. This part of the analysis focuses on understanding changes in the overall extent of suitable areas, shifts in spatial patterns, and the movement of the distribution centroid. This analysis will be accompanied by a comparative evaluation of prediction consistency and uncertainty among the various individual models, including GAM, GBM, RF, and MaxEnt. After subjecting the model outputs to rigorous accuracy assessments, this study integrates these findings to clarify how *C. barometz* responds ecologically to shifting climatic conditions. The insights gained are intended to offer a scientifically grounded framework to inform practical conservation decisions, including habitat restoration, guided cultivation and introduction efforts, and strategies for promoting the sustainable use of this species and the long-term protection of its populations.

## 2. Materials and Methods

### 2.1. Species Data Collection and Processing

**Figure 1:**
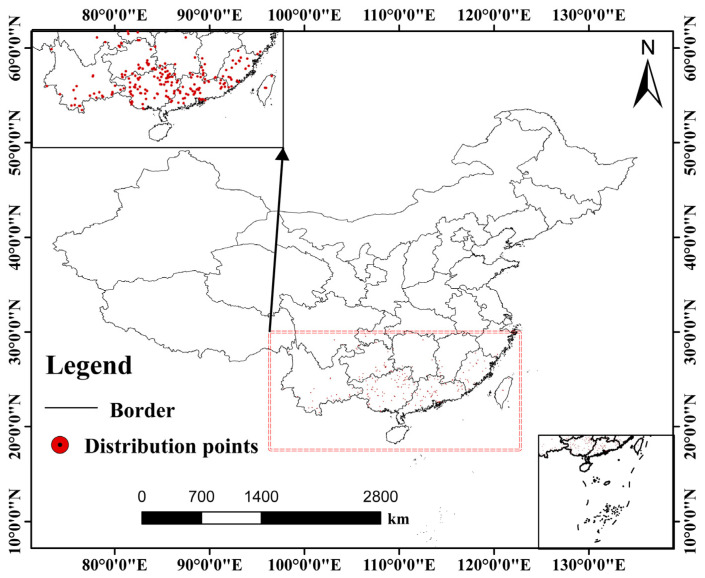
The distribution information of *C. barometz*.

Occurrence data for *C. barometz* were gathered from three authoritative sources, resulting in a total of 324 records collected between June 1869 and January 2025. These records were obtained from the Chinese Virtual Herbarium (CVH, http://www.cvh.ac.cn, accessed on 22 July 2025), the National Specimen Information Infrastructure (NSII, http://www.nsii.org.cn/2017/home.php, accessed on 25 July 2025), and the Global Biodiversity Information Facility (GBIF, https://www.gbif.org/, accessed on 28 July 2025) [37]. All records were verified to be unique and were required to possess precise geographic coordinates. Following the verification of unique records and geographic coordinates, all occurrence data were organized into a comma-separated values file and subsequently loaded into ArcGIS 10.8 (ESRI, Redlands, CA, USA) to support spatial analysis. Given the inherent clustered distribution pattern characteristic of *C. barometz*, which results in spatially aggregated occurrence data [38,39], a spatial filtering procedure was implemented to mitigate potential biases in the predictive modeling arising from this clustering effect. To minimize potential bias caused by spatial clustering in the occurrence data, we applied the “Spatially Rarefy Occurrence Data for SDMs” tool available in the SDM Toolbox. This procedure filtered the dataset so that no two remaining records were located within five kilometers of each other, a step that helped reduce issues related to spatial autocorrelation [40,41]. After applying this spatial filtering step, a total of 210 spatially independent occurrence points were retained and used in subsequent analyses, as shown in Figure 1. This spatial filtering (5 km thinning) is a form of spatial thinning designed to reduce geographic clustering and spatial autocorrelation [42]. Although species distribution models typically assume equilibrium between species and environment, *C. barometz* is a long-lived perennial fern with a relatively stable distribution over the historical period covered by our data. Retaining historical records since 1869 helps capture a more complete environmental gradient, thereby improving the environmental representativeness of the model. Furthermore, to assess the influence of temporal span on model predictions, we conducted a sensitivity analysis using a temporally filtered occurrence dataset (retaining only records after 1995) with the same parameter settings as the main model. The results, which are presented in Supplementary Table S9 and Supplementary Figures S9, S10 and S12, were used to validate the robustness of our core conclusions. Our use of a multi-model ensemble and spatial filtering further helps mitigate potential biases. Finally, the primary data were drawn from specimen records, which typically do not indicate whether individuals originated from wild or cultivated sources. Therefore, the final dataset may include both types. Including cultivated records may violate the natural niche assumption of SDMs, as cultivated individuals may persist in naturally unsuitable areas through artificial management (e.g., irrigation, shading), potentially distorting niche estimation [43]. To mitigate this potential bias, we applied spatial filtering (5 km thinning), a multi-model ensemble (RF, GAM, GBM, MaxEnt), and validated ecological plausibility through response curves and threshold analysis. Nevertheless, we recommend that future studies explicitly record source information or use independent datasets of wild occurrences for validation. To address other potential biases commonly found in herbarium records, such as the tendency for sampling sites to cluster near accessible locations, several precautions were incorporated into the study design [44]. To build a more generalized understanding of the relationship between the species and its environment, a series of coordinated steps was taken. A large number of pseudo-absence points were first drawn randomly from across the broader study area in China, ensuring broad environmental coverage and helping to reduce biases associated with sample accessibility. According to Barbet-Massin et al. [42], random pseudo-absence selection performs best for regression-based models (e.g., GLM, GAM), whereas environmentally or geographically stratified sampling may be preferable for classification and machine-learning methods. Because our study uses a multi-model ensemble that includes regression, classification, and machine-learning algorithms, random pseudo-absence generation is reasonable within this ensemble framework. The background selection area was restricted to the administrative boundary of China, corresponding to the accessible area M in the BAM framework. In addition, a multi-model ensemble approach was adopted, bringing together algorithms such as Random Forest and Generalized Additive Models. Because these methods differ in their sensitivity to various forms of bias, combining them helped counterbalance the tendency of any single model to overfit, thereby improving overall predictive reliability. Variable importance was also carefully assessed, and Pearson correlation filtering was applied to confirm that the environmental predictors retained were ecologically meaningful rather than artifacts of sampling patterns. Together, these measures were intended to support a more robust projection of the potential distribution of *C. barometz*. It should be noted that species distribution models typically assume that the species is in equilibrium with its environment, i.e., that its current distribution reflects climatic suitability [45,46]. Our predictions are based on this standard assumption. However, as *C. barometz* has been subject to long-term harvesting and habitat fragmentation, its current distribution may not fully reflect climatic suitability, particularly in historically over-harvested areas. Therefore, our projections should be interpreted cautiously under the equilibrium assumption, and potential biases are further discussed in Section 4.

### 2.2. Environmental Variable Selection and Climate Scenario Configuration

In order to assess how future climate conditions may shape the potential distribution of *C. barometz*, this study drew on a set of environmental variables closely tied to the species’ habitat preferences. These were grouped into three main types, namely bioclimatic factors, edaphic properties, and topographic characteristics, as summarized in Table 1. Current (1970–2000) and future bioclimatic data were obtained from the WorldClim database (http://www.worldclim.org/, accessed 8 October 2023) at a spatial resolution of 2.5 arc-minutes. For future projections under the Coupled Model Intercomparison Project Phase 6 (CMIP6) framework, we selected the BCC-CSM2-MR global climate model, which is known for its reliable performance in simulating extreme temperatures and precipitation patterns across China. To capture a broad spectrum of radiative forcing levels, the analysis incorporated four Shared Socioeconomic Pathways, namely SSP126, SSP245, SSP370, and SSP585, which represent a sustainability oriented trajectory, an intermediate development route, a regional rivalry scenario, and a fossil fuel dependent pathway, respectively [47]. The projection periods involved in this study include the mid-21st century, referring to the years 2041 to 2060, and the late 21st century, covering 2081 to 2100. Soil related data and topographic variables were, respectively, obtained from the Harmonized World Soil Database, accessed in November 2023, and the WorldClim database, accessed in October 2023. To maintain spatial consistency throughout the analysis, all environmental layers were subsequently clipped to match the administrative boundaries of China.

It should be noted that this study used only one GCM (BCC-CSM2-MR). Although this model performs well in simulating extreme climate over China, a single GCM cannot fully capture the uncertainty inherent in climate projections [48]. Therefore, our predictions should be interpreted as scenario-based trend estimates under this specific GCM rather than deterministic conclusions from a multi-model ensemble. This limitation is further discussed in Section 4.

To reduce the influence of collinearity among predictors on model stability, a multi-step screening strategy was implemented. Based on 210 occurrence records of *C. barometz* and an initial set of 37 environmental variables, we first assessed the contribution of each variable using models such as MaxEnt, and variables with zero or negligible contributions were excluded; this step was used only for initial screening and was not the sole criterion for final retention. Subsequently, environmental values at the occurrence points were extracted using ArcGIS, and Pearson correlation analysis was performed in SPSS 26. Given that the environmental variables (e.g., precipitation, temperature) are continuous and passed the Shapiro–Wilk normality test (*p* > 0.05), Pearson correlation is appropriate for such data according to Suherman et al. and Hauke & Kossowski [49,50]. To validate robustness, we additionally calculated Spearman’s rank correlation coefficients, which yielded highly consistent results for key variable pairs and did not alter the variable selection outcome. For variable pairs with |r| > 0.8, one variable was retained based on ecological relevance, literature support, and consistent performance across multiple models (e.g., RF, GAM). To further control for multicollinearity, Variance Inflation Factor (VIF) was calculated for all candidate variables, and VIF < 5 was set as the diagnostic criterion for variable retention. Detailed VIF results are presented in the Results Section (Section 3) and Supplementary Materials (Supplementary Table S1). This process resulted in 12 key environmental predictors for subsequent modeling. This strategy minimizes algorithm-specific biases and enhances the robustness and ecological relevance of variable selection [51].

### 2.3. Model Construction, Optimization and Evaluation

**Figure 2:**
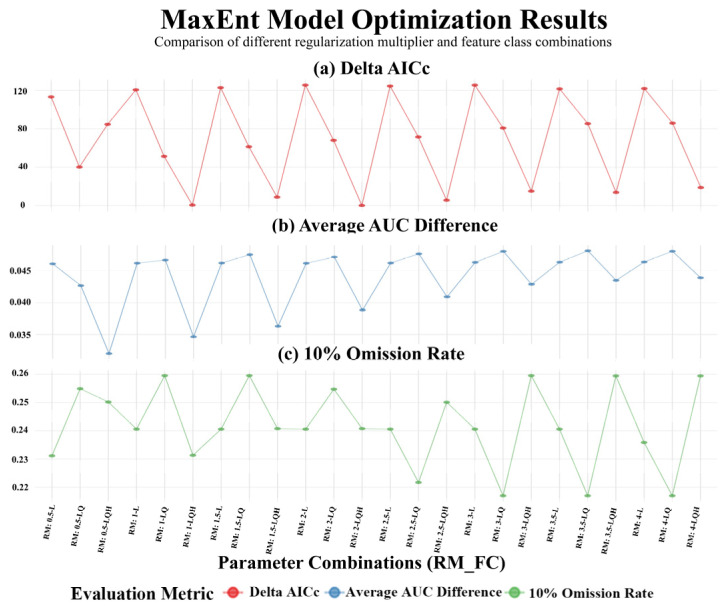
Optimization of the Maxent model: (**a**) delta AICc; (**b**) average AUC difference; (**c**) 10% omission rate.

To model the potential distribution of *C. barometz*, we used MaxEnt software version 3.4.4 (AMNH, New York, NY, USA). Model optimization and all subsequent analyses were carried out using the refined set of key environmental variables obtained from the screening process described in Section 2.2. In order to improve the model’s ability to generalize and to reduce the likelihood of overfitting, we optimized its critical parameters with the ENMeval package version 2.0.5.2 (CRAN, https://cran.r-project.org/web/packages/ENMeval/, accessed on 21 March 2026) in RStudio 4.4.1 (PBC, Boston, MA, USA). The regularization multiplier was systematically calibrated by testing values ranging from 0.5 to 4.0 in increments of 0.5, while feature class combinations including L, LQ, H, LQH, LQHP, and LQHPT were also evaluated. The most appropriate parameter set was determined by identifying the configuration that minimized the corrected Akaike Information Criterion, reflected by the lowest delta AICc value [44,52]. As part of the model development process, we applied this parameter optimization to the refined set of environmental variables that had already undergone screening to ensure they were non-redundant. The outcomes of this tuning procedure are illustrated in Figure 2. For all subsequent model runs, we established consistent baseline settings in which 75 percent of the occurrence points were randomly assigned to the training group and the remaining 25 percent were set aside for model validation. To support stable model convergence in this complex ecological analysis, the maximum number of iterations was set to 1,000,000, a commonly used upper limit that helps ensure adequate model fitting. In practice, however, the actual runtime is guided by a convergence threshold set at 1.0 × 10^−5^, meaning that the model stops iterating once this criterion is met, which helps avoid premature termination and reduces the risk of unstable predictions. The post-optimization difference between training and validation AUC was only 0.0147, indicating good convergence. In addition, a 10-fold cross-validation approach was used to validate model performance, and the contribution of each environmental variable was also evaluated using a jackknife test. MaxEnt by default applies a “clamping” function, which restricts predictions for variable values outside the training data range to the nearest boundary value (minimum or maximum), thereby avoiding unconstrained extrapolation [53]. This setting ensures that future climate projections do not extend beyond the environmental space of the training data, reducing extrapolation uncertainty. To account for spatial autocorrelation in occurrence data, we additionally performed spatial block cross-validation [54,55] alongside the conventional 10-fold random cross-validation. Using the blockCV R package version 3.2.0 (CRAN, https://cran.r-project.org/web/packages/blockCV/, accessed on 21 March 2026), we partitioned the 210 occurrence points into four spatially independent blocks based on spatial clustering of their geographic coordinates. Each block in turn served as the validation set, with the remaining blocks used for training, repeated four times per model. The AUC results from spatial block cross-validation are reported alongside the random cross-validation results in Supplementary Table S5. To assess model performance, we primarily relied on the Area Under the Receiver Operating Characteristic Curve, commonly referred to as the AUC. According to established standards, a value of 0.8 or higher reflects reliable predictive ability, while scores falling between 0.9 and 1.0 are considered indicative of high predictive accuracy [56]. It should be noted that relying solely on AUC has limitations [57]. Therefore, we also used TSS, Kappa, and the Continuous Boyce Index (CBI) as complementary evaluation metrics (see Section 3.1 and Supplementary Table S4). CBI is a presence-only calibration metric that is insensitive to pseudo-absence selection. After completing model optimization, we used it to project potentially suitable habitats for *C. barometz* under current climate conditions as well as for the 2050s and 2090s under four future climate scenarios, namely SSP126, SSP245, SSP370, and SSP585.

To ensure consistency in how habitat suitability was classified across different time periods and climate scenarios, this study applied the Natural Breaks classification method consistently [44]. This technique groups data by maximizing the differences between classes according to the natural distribution of values within each raster dataset. Doing so helps prevent the subjective bias that can result from using a fixed or arbitrarily chosen threshold for classification. This classification approach, grounded in the natural breaks of the dataset, ensures that categories such as highly suitable areas are defined in a way that aligns with the underlying structure of the data across a given time and space. As a result, the ecological meaning of each suitability class becomes more transparent and interpretable. Maintaining this methodological consistency is paramount for cross-scenario comparisons. Although the absolute probability values associated with habitat suitability may differ among various climate scenarios, maintaining a consistent classification algorithm allows comparisons to focus on spatial dynamics. This includes shifts in the distribution centroid as well as expansions or contractions of areas identified as relatively highly suitable according to a uniform standard. This approach helps ensure that any observed trends genuinely stem from shifts in climatic conditions rather than from artificial discrepancies introduced by inconsistent classification techniques. By maintaining this consistency, the study establishes a reliable basis for comparing results across different timeframes and emission scenarios [51,56]. It should be noted that Natural Breaks is a data-driven classification method, and the resulting suitability categories are defined based on the internal distribution of each raster dataset. Consequently, these categories are not strictly comparable across different climate scenarios in an absolute ecological sense. Therefore, our cross-scenario comparisons focus on relative spatial patterns within each scenario (e.g., direction and distance of centroid shifts, fragmentation trends) and qualitative comparisons of change trajectories across scenarios, rather than absolute area comparisons of suitability categories. All conclusions are drawn with this cautious premise. Furthermore, to test the robustness of our core conclusions to the choice of classification method, we conducted a sensitivity analysis using a fixed threshold (10th percentile of training presence). This threshold was extracted from the optimized MaxEnt model with 10-fold cross-validation, yielding a mean Cloglog value of 0.3927. After uniformly applying this threshold across all scenarios, we recalculated centroid shift directions and suitable area changes. The results showed that the core conclusions—for example, a southwestward centroid shift by the 2090s under SSP585 and the trends in suitable area changes—were highly consistent with those obtained using Natural Breaks (see Supplementary Table S10), indicating that our main findings are insensitive to the choice of classification method.

To better account for predictive uncertainty and improve the reliability of the results, we built a multi-model ensemble framework that initially integrated nine algorithms, namely GLM, GAM, GBM, CTA, ANN, SRE, MARS, and RF. Using the Biomod2 package version 4.2.6.2 (CRAN, https://cran.r-project.org/web/packages/biomod2/, accessed on 21 March 2026) in RStudio 4.4.1, we implemented these eight algorithms, excluding any that failed to reach convergence during model runs [44,52]. Each algorithm was executed with ten replicates, based on a random partition of the data with seventy-five percent used for training and the remaining twenty-five percent reserved for validation. To improve the models’ capacity to distinguish between suitable and unsuitable conditions, one thousand pseudo-absence points were also generated. Each algorithm was used with its default robust parameter settings within Biomod2: RF with ntree = 500; GBM with n.trees = 1000, interaction.depth = 3, shrinkage = 0.01; GAM with default degrees of freedom from mgcv; GLM with stepwise selection based on AIC; CTA with complexity parameter cp = 0.01; ANN with size = 2 and decay = 0.01; MARS with degree = 2 and nk = 10; and SRE as an envelope model. All models were run with 75% training and 25% testing data, 1000 randomly generated pseudo-absence points, and 10 repeated runs (5 runs for ANN due to convergence constraints). Detailed parameter settings are provided in Supplementary Table S3.

To evaluate model performance comprehensively, we applied the True Skill Statistic, the Kappa coefficient, and the AUC value. It should be noted that TSS, Kappa, and AUC are threshold-dependent or pseudo-absence-dependent metrics, which may yield overly optimistic performance estimates when applied to presence-only data due to the choice of pseudo-absences. To address this limitation, we additionally used the Continuous Boyce Index (CBI) as a presence-only evaluation metric [58]. CBI partitions the predicted habitat suitability range into moving windows, calculates the predicted-to-expected (P/E) ratio of presence points within each window, and uses Spearman’s rank correlation to assess the consistency between predicted suitability and presence distribution. CBI does not rely on pseudo-absences and better reflects model predictive capability for presence-only data. Only models meeting the established thresholds, specifically an AUC of at least 0.80, a TSS of 0.60 or higher, and a Kappa value of 0.50 or above, were considered well-performing and retained for further analysis [59,60]. The final ensemble projection was then developed through a two-stage procedure. Within the Biomod2 platform, individual models that successfully met all three predefined performance criteria were first selected. Their predictions were then integrated with the output from the independently optimized MaxEnt model mentioned earlier using equal-weight averaging to generate a consensus ensemble projection (see Supplementary Table S6). It should be noted that MaxEnt was tuned separately using ENMeval because the current version of Biomod2 does not support such fine-scale automated parameter tuning internally; this is a technical compromise rather than an attempt to assign higher weight to MaxEnt. All models meeting the performance thresholds (including the optimized MaxEnt) contributed equally to the final prediction. To determine the optimal ensemble strategy, we compared equal-weight averaging, weighted averaging (based on TSS), and consensus voting (see Supplementary Table S6), selecting the best-performing method as the final ensemble approach. Only those models fulfilling every evaluation threshold were retained in the final ensemble. The resulting consensus probability surface was subsequently reclassified into four habitat suitability levels, namely unsuitable, low suitability, moderate suitability, and high suitability, once again applying the Natural Breaks method. Through systematic comparisons of both the extent of suitable areas and the shifts in spatial patterns across various climate scenarios and timeframes, this integrated modeling approach provides a clearer understanding of how future climate change may influence the potential distribution of *C. barometz*.

### 2.4. Centroid Migration Analysis

To evaluate how suitable habitats may shift spatially in response to projected climate change, we calculated the geometric centroid of the predicted distribution area for *C. barometz*. This was carried out using the Mean Center tool within the Spatial Statistics module of ArcGIS 10.8, following established methodologies [44,61]. Centroids were generated for each time period examined, including the current baseline, the 2050s, and the 2090s, and were computed separately for every future climate scenario included in the analysis. This quantitative assessment was intended to elucidate the species’ spatial response trend and migration trajectory, thereby providing insights into how *C. barometz* may adjust its distributional range in geographical space as a consequence of ongoing climatic shifts.

## 3. Results

### 3.1. Model Parameter Optimization and Performance Evaluation

After identifying the key environmental variables outlined in Section 2.2, we proceeded to optimize the MaxEnt model using the ENMeval package version 2.0.5.2, focusing on two critical parameters: the regularization multiplier and the feature class combination. A range of configurations was tested, and the optimal settings were selected based on the lowest delta AICc value. As shown in Figure 2, the combination that yielded the best performance consisted of a regularization multiplier of 2 and a feature class of LQH. This combination of parameters yielded a delta AICc value of zero, indicating that an optimal tradeoff between model fit and complexity was achieved. The final model exhibited strong predictive performance and stability, with a mean validation AUC of 0.939, an average AUC difference of 0.0388, and a ten percent test omission rate of 0.241. The advantages of this optimization process were evident when compared against the default parameter settings, which used a regularization multiplier of one and automatic feature selection. Under the optimized configuration, the number of coefficients in the model decreased from 44 to 28. More notably, the gap in AUC values between the training and validation datasets was reduced from 0.0178 to 0.0147, suggesting that overfitting was effectively controlled and that the model’s capacity for generalization and ecological interpretation had improved. As a result, this set of parameters was applied consistently across all subsequent simulations of habitat suitability.

Following this evaluation, the predictive performance of all eight algorithms was compared and assessed in detail (Supplementary Figure S1, Table 2). Among them, RF, GAM, and GBM showed strong and consistent predictive performance, with AUC values exceeding 0.90 and both TSS and Kappa scores above 0.60. These three algorithms met the preset performance threshold criteria (AUC ≥ 0.80, TSS ≥ 0.60, Kappa ≥ 0.50). In addition, the independently optimized MaxEnt model achieved a mean test AUC of 0.950 (SD 0.009) from 10-fold cross-validation, also meeting the threshold criteria. Therefore, a total of four algorithms (RF, GAM, GBM, and the optimized MaxEnt) were selected for subsequent equal-weight ensemble modeling. To further validate the predictive capability of the models on presence-only data, we calculated the Continuous Boyce Index (CBI) for RF, GAM, GBM, and the optimized MaxEnt; the results are summarized in Supplementary Table S4. RF had a CBI of 0.992, GAM 0.973, GBM 0.956, and the optimized MaxEnt 0.992, all exceeding 0.95, indicating excellent predictive performance on presence-only data and robust model reliability.

To assess the impact of spatial autocorrelation on model performance, we performed spatial block cross-validation by partitioning the 210 occurrence points into four spatially independent blocks using the blockCV R package, with each block serving sequentially as the validation set in a 4-fold cross-validation. As shown in Supplementary Table S5, the resulting mean AUC values were 0.9639 (SD 0.0046) for RF, 0.9405 (SD 0.0120) for GAM, 0.9506 (SD 0.0074) for GBM, and 0.9516 (SD 0.0053) for the optimized MaxEnt. These values were almost identical to those obtained from random cross-validation (RF: 0.9643; GAM: 0.9396; GBM: 0.9519; MaxEnt: 0.9531), with a maximum difference of less than 0.002. This indicates that our models are insensitive to spatial data partitioning strategies and remain highly robust under spatially independent validation.

It should be noted with caution that the above AUC, TSS, and Kappa values were obtained in the context of randomly generated pseudo-absences, and these metrics may vary to some extent depending on pseudo-absence selection. Nevertheless, combined with the robust CBI results (all >0.95) and the multi-model ensemble strategy, we consider our model performance evaluation to be reliable. Their selection reflects their ability to reliably capture the distribution patterns of *C. barometz* and confirms their appropriateness for modeling habitat suitability in this study.

### 3.2. Identification of Dominant Environmental Factors and Analysis of Suitable Thresholds

Following an initial assessment that considered variable contribution values from the MaxEnt model along with Pearson correlation analysis and variance inflation factor testing, twelve key environmental predictors were retained from the original thirty-seven for use in the final modeling process. To validate the robustness of the correlation analysis, we additionally calculated Spearman’s rank correlation coefficients and generated a correlation heatmap (Supplementary Figure S2). The results showed that the correlation structure of key variable pairs was highly consistent with that obtained using Pearson, and the variable selection outcome remained unchanged. The optimized MaxEnt model, which was configured with a regularization multiplier of two and a feature class of LQH, showed strong predictive performance. A tenfold cross validation produced a mean test AUC of 0.950 with a standard deviation of 0.009, indicating both high accuracy and stability in the model’s performance. This outcome demonstrates the model’s strong predictive capability and reliable stability, achieving a sound balance between explanatory strength and model simplicity while minimizing the potential for overfitting. To gain deeper insight into which environmental factors most strongly shaped the distribution of *C. barometz*, we examined both the percent contribution and the permutation importance for each variable within the optimized modeling framework. In terms of percent contribution, Bio17 was the most influential variable, accounting for 52.6% of the model’s explanatory power. This was followed by Bio12 at 24.9% and Bio6 at 10.2%. Together, these three climatic factors contributed 87.7%, highlighting their central role in shaping the distribution of *C. barometz*. A different but complementary perspective emerged from the permutation importance analysis, where Bio7 ranked highest at 45.9%, again followed by Bio6 at 22.3% and Bio17 at 10.4%. This metric further underscores the combined influence of temperature and water availability in limiting the species’ range. By comparison, edaphic and topographic variables such as S_oc, slope, and T_silt each contributed less than 3%, indicating that these factors play a secondary, modulating role rather than serving as primary drivers of broad-scale distribution patterns. Taken together, these findings highlight the predominant influence of hydrothermal conditions, particularly dry-season precipitation and low-temperature stress, in defining the ecological niche of *C. barometz*.

**Figure 3:**
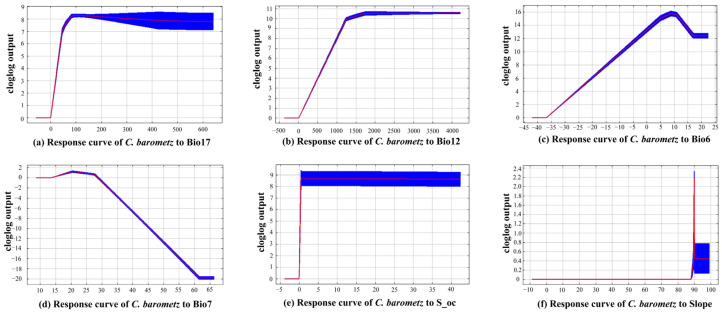
The response curve of dominant environmental factors: (**a**) Bio17 Precipitation of the driest quarter; (**b**) Bio12 Annual precipitation; (**c**) Bio6 Minimum temperature of the coldest month; (**d**) Bio7 Temperature annual range; (**e**) S_oc Subsoil organic carbon content; (**f**) Slope. Red and blue curves represent the response based on training and test data, respectively.

An examination of the response curves (Figure 3) elucidates the complex, nonlinear relationships between *C. barometz* and its core environmental determinants, while also revealing the specific thresholds at which conditions become suitable (defined here as a presence probability exceeding 0.5). As shown in Table 3, the four variables with the greatest influence on the model, namely Bio17, Bio12, Bio6, and Bio7, together explain 94% of the variation, clearly identifying them as the primary climatic drivers shaping the distribution of *C. barometz*. By comparison, edaphic factors such as S_oc and topographic variables like Slope make relatively minor contributions, functioning instead as secondary influences that fine-tune habitat suitability rather than determining broad-scale patterns. Consequently, a detailed threshold analysis was undertaken, focusing specifically on these primary drivers, with the results systematically presented in Table 4. It should be noted that the “suitable range” in Table 4 represents the entire interval where predicted occurrence probability > 0.5. The extreme values (e.g., the lower limit of 3.25 mm for Bio17, −35.41 °C for Bio6) are mathematical boundaries arising from the asymptotic nature of the fitted response curves and do not imply actual ecological tolerance. In contrast, the “adaptive threshold” is the ecologically meaningful indicator.

As shown in Table 4, the distribution of *C. barometz* is closely tied to warm and humid climatic conditions. For Bio17, the species can tolerate precipitation levels ranging from 3.25 to 640.20 mm, although optimal growth requires values above 96.84 mm. Annual precipitation, represented by Bio12, falls within a suitable range of 74.58 to 4209.60 mm, with the most vigorous growth occurring when precipitation reaches between 3834.10 and 4209.60 mm, highlighting a clear need for abundant moisture. Sensitivity to low temperatures is also evident from the response to Bio6; while the tolerated range spans from 35.41 °C to 22.35 °C, optimal conditions demand that temperatures remain above 8.79 °C. In addition, although *C. barometz* can withstand a fair degree of seasonal temperature variation, it shows a preference for thermal stability, with suitable Bio7 values ranging from 16.25 °C to 27.92 °C and an optimal value around 20.47 °C.

In addition to the main climatic influences, soil properties such as organic carbon content and terrain features like slope also contribute to shaping habitat suitability. The suitable range for S_oc falls between 0.03% and 42.30%, with optimal conditions occurring when values exceed 0.49%. Slope analysis reveals a highly constrained suitable range from 89.60° to 90.03°, with an optimum near 89.92°, indicating that this species is closely associated with steep mountainous terrain, a pattern consistent with the topographic characteristics of its known distribution areas. As seen in the response curves (Figure 3), the predicted probability approaches zero at these extreme values; therefore, interpretation should focus on the adaptive thresholds. Taken together, the potential range of *C. barometz* is largely shaped by the combined effects of water availability and low temperature stress. As a result, its core habitats closely correspond to subtropical humid monsoon regions characterized by high rainfall, mild winters, and undulating hilly or mountainous topography.

To further validate the statistical robustness of our variable selection, we performed Variance Inflation Factor (VIF) multicollinearity diagnostics on the 12 retained variables. The results showed that Bio5 and Bio17 were automatically excluded from the linear model due to perfect collinearity with other variables, while the remaining 10 variables had VIF values below 5 (range 1.031–4.102; see Supplementary Table S1), indicating acceptable multicollinearity. Although Bio5 and Bio17 were not included in the VIF calculation, they were retained based on their high contribution rates in the MaxEnt model (Bio17: 52.6%, Bio5: 0.5%) and clear ecological significance (precipitation of the driest quarter and maximum temperature of the warmest month).

Furthermore, to assess the stability of variable importance across different models, we integrated eight algorithms (RF, GBM, MARS, GAM, GLM, CTA, ANN, SRE) within the Biomod2 framework, each run 10 times. The summary of variable importance (see Supplementary Table S2) showed that Bio9 (mean temperature of the driest quarter), Bio14 (precipitation of the driest month), Bio13 (precipitation of the wettest month), Bio15 (precipitation seasonality), Bio2 (mean diurnal temperature range), Bio8 (mean temperature of the wettest quarter), as well as soil properties including T_sand, T_ece, and S_silt, exhibited consistently high importance across multiple models, further supporting the reliability of our variable selection.

### 3.3. Potential Distribution Pattern Under Current Climatic Conditions

**Figure 4:**
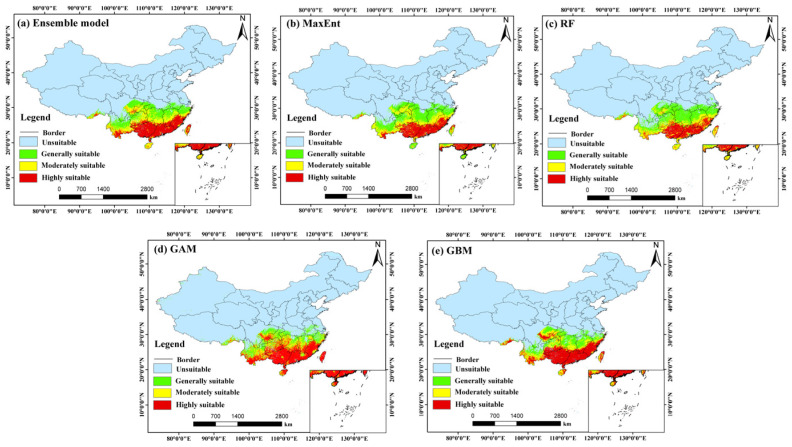
Potential distribution of *C. barometz* under present climatic conditions predicted by different modeling approaches. (**a**) Ensemble model; (**b**) MaxEnt; (**c**) RF; (**d**) GAM; (**e**) GBM.

Based on the ensemble model outputs, the potential distribution of *C. barometz* under current climate conditions was mapped and analyzed, as shown in Figure 4. To determine the optimal ensemble strategy, we compared three methods: equal-weight averaging, weighted averaging (based on TSS), and consensus voting. The results (Supplementary Table S6) showed that equal-weight averaging achieved the highest performance (AUC = 0.9641, TSS = 0.8420, Kappa = 0.7944), outperforming the other two methods. Therefore, equal-weight averaging was selected as the final ensemble approach. The results indicate that suitable habitats are largely located in areas south of the Yangtze River Basin, with a clear latitudinal pattern that reflects the species’ strong preference for the warm and humid environments typical of subtropical regions. The ensemble model exhibited strong predictive performance and stability, achieving high evaluation scores with a TSS of 0.842, an AUC of 0.964, and a Kappa value of 0.794. In terms of spatial distribution, both highly suitable and moderately suitable areas together form the core ecological range of *C. barometz*. These areas are mainly found across a broad belt stretching from southeastern Tibet through southern Yunnan, southeastern Sichuan, and southwestern Chongqing, continuing into southern Guizhou, Guangxi, Guangdong, and southern Jiangxi, and extending further to Fujian, southern Zhejiang, Taiwan, and southern Hainan. With abundant hydrothermal conditions and favorable terrain, these locations offer ideal environments for the species to grow and become established. By comparison, areas of general suitability reach farther north, covering parts of eastern Sichuan, northern Chongqing, northern Guizhou, southern Hubei, Hunan, northern Jiangxi, southern Anhui, and northern Zhejiang, forming the outer edges of the species’ potential range. According to current estimates, the total suitable habitat for *C. barometz* covers roughly 170.67 × 10^4^ km^2^, which corresponds to about 17.78% of China’s total land area. Within this region, highly suitable areas make up 57.30 × 10^4^ km^2^, equivalent to 5.97% of the country, while moderately suitable areas account for 51.12 × 10^4^ km^2^ or 5.33%. Together, these figures reflect the species’ strong preference for particular microenvironments rather than a uniform occupation of all climatically favorable zones.

When comparing the outputs of individual models, the spatial patterns generated by MaxEnt, RF, GAM, and GBM showed strong agreement with the ensemble model results. All of these approaches consistently identified the humid provinces of southern China as the primary areas of habitat suitability, as illustrated in Figure 4b–e. The total suitable areas projected by these individual models were 160.76 × 10^4^ km^2^ (MaxEnt), 163.48 × 10^4^ km^2^ (RF), 166.91 × 10^4^ km^2^ (GAM), and 157.33 × 10^4^ km^2^ (GBM), all of which align closely with the ensemble model’s estimate of 170.67 × 10^4^ km^2^. Notably, the GAM and GBM models predicted relatively larger extents of highly suitable areas, at 74.95 × 10^4^ km^2^ and 69.64 × 10^4^ km^2^, respectively. In contrast, the MaxEnt and RF models yielded comparatively smaller estimates for this category, at 42.94 × 10^4^ km^2^ and 39.78 × 10^4^ km^2^, respectively. These discrepancies reflect the inherent sensitivity of different algorithms in delineating core habitat extents. Nevertheless, all models consistently corroborated the predominant status of the South China to Southwest China region as the primary suitable zone for *C. barometz*. By offsetting the algorithm-specific biases inherent in individual predictions, the ensemble model ultimately yields a distribution estimate that is both more conservative and more consistent with ecological expectations. This makes it the most suitable reference point for guiding subsequent analytical steps.

### 3.4. Projected Potential Distribution Under Future Climate Scenarios

**Figure 5:**
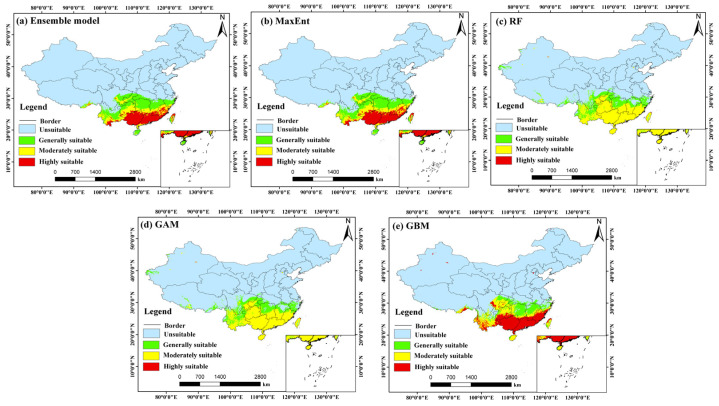
Potential suitable habitats of *C. barometz* predicted by different models under the SSP126 scenario for the 2050s: (**a**) Ensemble model; (**b**) MaxEnt; (**c**) RF; (**d**) GAM; (**e**) GBM.

**Figure 6:**
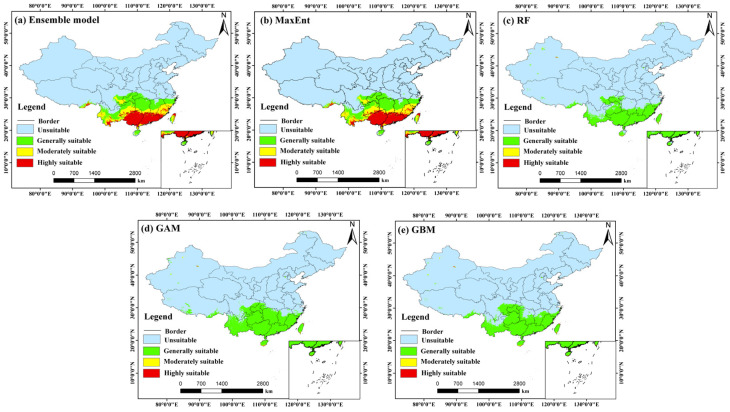
Potential suitable habitats of *C. barometz* predicted by different models under the SSP585 scenario for the 2090s: (**a**) Ensemble model; (**b**) MaxEnt; (**c**) RF; (**d**) GAM; (**e**) GBM.

Using an ensemble of five species distribution models, including MaxEnt, Random Forest, Generalized Additive Model, Gradient Boosting Machine, and a combined ensemble approach, this study examined the complex responses of potential suitable habitats for *C. barometz* under future climate scenarios. Despite inherent algorithmic differences, these models collectively revealed a degree of consensus regarding spatiotemporal evolution patterns across various Shared Socioeconomic Pathways (SSPs), as detailed in Figure 5 and Supplementary Figures S3–S8, as well as Figure 6. By bringing together the projections generated by these well-performing individual algorithms, the ensemble model produced a spatially consistent and integrated picture of habitat suitability, which further supports the credibility of the overall patterns observed.

**Figure 7:**
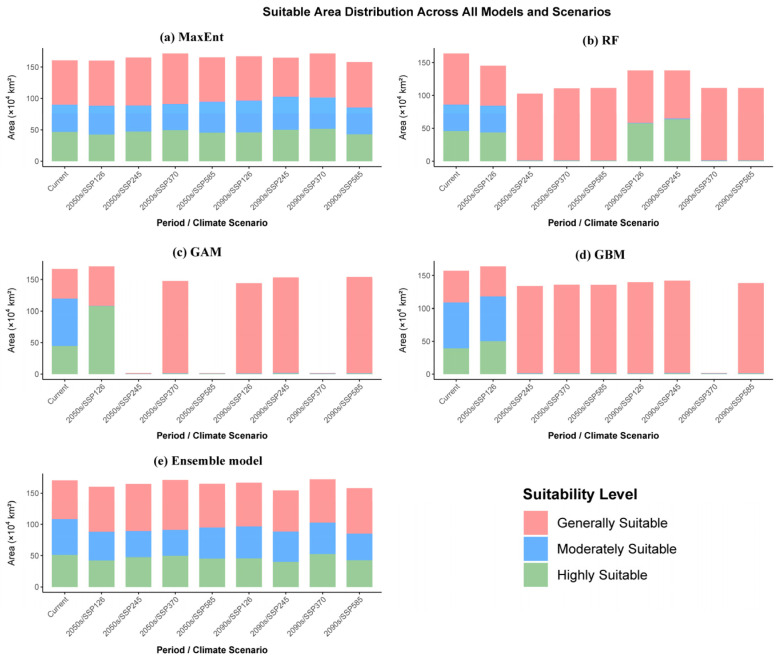
Suitable area of *C. barometz* under different future climate scenarios and periods: (**a**) MaxEnt; (**b**) RF; (**c**) GAM; (**d**) GBM; (**e**) Ensemble model.

Based on the simulation results, it is evident that the trajectory of greenhouse gas emissions will significantly alter the distribution of suitable habitat for *C. barometz*. Under the low emission pathway represented by SSP126, the species appears to possess a relatively high degree of adaptive capacity. According to the ensemble model projections, the total area of suitable habitat is expected to undergo a modest decline of approximately 5.98% by the 2050s, after which it shows signs of leveling off, with only a slight further reduction of 2.17% by the 2090s, as illustrated in Figure 5a. This pattern of relative stability was corroborated by individual models such as MaxEnt, which displayed analogous trends (Figure 5b–e). These results suggest that *C. barometz* may retain relatively stable habitat conditions under moderate climatic shifts. By contrast, the high emission SSP585 pathway presents considerably greater challenges for the species. While certain algorithms such as GAM indicated some short term variability in suitable area during the 2050s, most models pointed to a substantial and sustained reduction by the 2090s as the cumulative effects of intensified greenhouse forcing become more pronounced. The Ensemble model similarly reflected this decline, showing a reduction in total suitable area ranging from 0.07% to 3.2% (Figure 6a). Under the high emission trajectory represented by SSP585, the areas identified as highly suitable for *C. barometz* showed notable reduction and isolation. This fragmentation was particularly evident in the projections generated by the Random Forest and Generalized Additive Models, both of which indicated that optimal habitats would shrink considerably and become mostly restricted to scattered pockets within Hainan and Taiwan. For example, RF predicted a reduction in total suitable area of 31.76%, whereas GAM predicted only 7.68%, reflecting inter-model uncertainty. Such outcomes imply that the severity of future climatic conditions could exceed what the species is physiologically able to tolerate. A comparative view of how suitable habitat areas are expected to evolve across different timeframes and emissions scenarios is presented in Figure 7, which synthesizes the projected changes in a way that highlights their divergence under varying climate pathways.

When viewed from a spatial perspective, the projected distribution of suitable habitats for *C. barometz* reveals a clear pattern of dynamic change. According to the ensemble model outputs, highly suitable areas are expected to remain largely confined to the species’ traditional core regions during the 2050s across all climate scenarios considered. These areas include southeastern Xizang, southern Yunnan, southeastern Sichuan, southwestern Chongqing, southern Guizhou, Guangxi, Guangdong, southern Jiangxi, Fujian, southern Zhejiang, and Taiwan, as illustrated in Figure 5a and Supplementary Figures S4a, S6a and S8a. By the 2090s, while the core areas persist predominantly in South and Southwest China, notable transformations are projected along the distributional margins. Under the low-emission SSP126 pathway, a clear northward expansion of moderately suitable habitat is projected, with suitable conditions extending into parts of eastern Sichuan, northern Chongqing, southern Hubei, northern Hunan, northern Jiangxi, southern Anhui, and even reaching northern Zhejiang. This movement reflects a broader ecological response in which species shift toward higher latitudes as temperatures rise. By contrast, under the high-emission SSP585 scenario, more intense thermal stress and changing precipitation patterns along the southeastern coast are expected to drive a contraction of highly suitable areas, which become increasingly concentrated in inland locations such as southeastern Yunnan, eastern Sichuan, and southwestern Chongqing. As a result, some historically core areas, including southern Guangdong, are anticipated to experience a decline in habitat suitability.

Taken together, these results highlight not only the significant impact that future climate change is likely to have on the geographic distribution of *C. barometz*, but also the value of proactive efforts to reduce emissions. Pathways such as SSP126, which emphasize sustainability, appear particularly favorable for supporting the long-term stability of its populations. Conversely, the high-emission SSP585 trajectory poses a substantial risk of core habitat loss and fragmentation. Given the consistency and reliability of the projections produced by our ensemble modeling approach, these consensus results offer a robust spatial evidence base to support regional conservation planning and inform the design of targeted climate adaptation measures. As a robustness check, the results obtained using the fixed threshold (10th percentile training presence threshold) were highly consistent with those from Natural Breaks in terms of core qualitative conclusions such as centroid shift direction (see Supplementary Table S10).

### 3.5. Spatiotemporal Dynamics of Centroid Shift in Suitable Habitats

**Figure 8:**
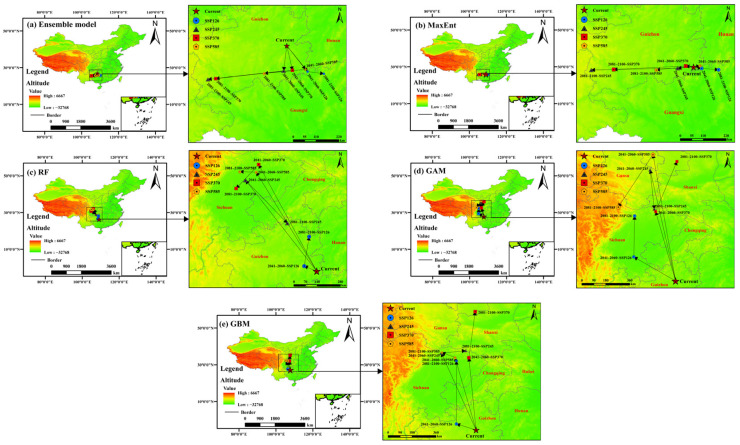
Centroid migration trajectories of the total suitable area for *C. barometz* under future climate scenarios: (**a**) Ensemble model; (**b**) MaxEnt; (**c**) RF; (**d**) GAM; (**e**) GBM.

Projections of future centroid shifts for suitable habitats of *C. barometz*, generated from five modeling approaches including MaxEnt, Random Forest, Generalized Additive Model, Gradient Boosting Machine, and an ensemble approach, point to a broadly consistent trend of southwestward movement, although the predicted routes and distances vary to some extent among the different algorithms as illustrated in Figure 8. This spatial pattern implies that in response to ongoing climate warming, the species may be retreating toward higher elevations or lower latitudes within inland southwestern areas, especially the eastern Yunnan-Guizhou Plateau and its surrounding zones, as it seeks out suitable climatic refugia.

When looking closely at results from each modeling approach, the centroid paths generated by MaxEnt, RF, and GAM all point to a continued southwestward movement from the present transitional zone between South and Southwest China toward Guizhou, Guangxi, and Yunnan. Taking the RF model as an example, it showed the most striking shift under the high emission SSP585 scenario, with a projected movement of 678.52 km by the 2050s. This was followed by a smaller adjustment of 84.08 km back in the opposite direction by the 2090s, a trajectory first marked by a strong westward movement, followed by a period of smaller shifts. Similarly, the GAM model demonstrated a strong response to this high-forcing scenario, with predicted centroid shifts of 967.33 km and 671.86 km for the 2050s and 2090s, respectively, its centroid moving significantly northward into the region north of the Qinling-Daba Mountains, thereby reflecting the algorithm’s heightened sensitivity to extreme climatic conditions. In contrast, the GBM model projected a relatively more moderate migration pathway, with a displacement of 564.59 km under the SSP585 scenario in the 2050s, which then receded to 92.75 km by the 2090s. Notably, its centroid remained predominantly within the eastern margins of the Yunnan-Guizhou Plateau, a finding that aligns closely with the overarching trend of southwestward contraction identified by the other models.

The ensemble model, by synthesizing the consensus predictions from the robust individual algorithms, offered a particularly compelling and representative portrayal of centroid migration trajectories (Figure 8a, Supplementary Table S8). Under current climatic conditions, the centroid was situated at the junction of Yunnan, Guizhou, and Guangxi provinces, specifically at coordinates 108.46° E, 26.99° N. Projecting forward to the 2050s, a consistent southward displacement of the centroid is evident across all SSP scenarios, with migration distances ranging from 120.9 km under SSP370 to 153.9 km under SSP126, resulting in a latitudinal shift to between 25.86° N and 25.96° N. By the 2090s, however, these migration pathways exhibit a marked divergence depending on the emissions trajectory. Under the relatively mild SSP126 scenario, the centroid continues its southeastward progression, moving an additional 71.2 km to settle at 110.07° E, 25.77° N. This stands in sharp contrast to the pronounced southwestward shift driven by the higher-emission pathways of SSP245, SSP370, and SSP585. Under these scenarios, the centroid migrates considerably further, with distances of 331.3 km, 335.1 km, and 180.2 km, respectively, ultimately concentrating within a longitudinal band of 105.04° E to 107.48° E and a stable latitudinal range of 25.52° N to 25.78° N. The differences observed among these projected pathways offer meaningful insight. They show how the directional trend captured by the ensemble model is largely consistent with the directional trends observed across the individual models, while also revealing that migration distances tend to increase under scenarios with stronger radiative forcing. Together, these patterns support the view that shifting southwestward represents a key spatial response by *C. barometz* in coping with growing climatic stress.

Overall, the simulated southwestward movement of suitable habitat centroids becomes increasingly evident under higher emission pathways, highlighting the strong influence of climatic factors in driving shifts in species distributions. The consistent projections generated by the ensemble model not only reinforce this observed pattern but also offer practical spatial guidance that can directly inform conservation planning and management decisions. They explicitly identify the southwestern regions of China, more precisely southeastern Yunnan, southern Guizhou, and western Guangxi, as critical areas that should be prioritized for future germplasm resource conservation, thereby informing strategic conservation planning and deployment efforts. It should be noted that the suitable habitat classification in this section is based on the Natural Breaks method. To test the robustness of this classification approach, we conducted a sensitivity analysis using a fixed threshold, specifically the 10th percentile training presence threshold with a Cloglog value of 0.3927 (Supplementary Figure S11). The results showed that under high-emission scenarios like SSP585, SSP245, and SSP370, the southwestward direction of centroid shift was fully consistent with that obtained using Natural Breaks. Under the low-emission SSP126 scenario, the southeastward shift direction was also consistent. The trends in suitable area changes were highly consistent as well (see Supplementary Table S10). This confirms that our core conclusion, namely that the suitable habitat of *C. barometz* contracts southwestward under high-emission scenarios, remains robust regardless of the choice of classification method.

## 4. Discussion

In this study, a multi-model ensemble approach was employed to systematically explore the potential geographical distribution and spatiotemporal dynamics of *C. barometz* under both contemporary and future climate scenarios. The findings reveal that suitable habitats for this species are predominantly concentrated within the warm, humid hilly and mountainous regions of southern China, encompassing Yunnan, Guangxi, Guangdong, Fujian, Hainan, and Taiwan, which collectively exhibit a notably fragmented distribution pattern. This spatial configuration aligns remarkably well with the documented natural occurrence of the species in tropical and subtropical shaded environments as recorded in the *Flora of China*, thereby corroborating its ecological characterization as a moisture-loving and shade-tolerant fern [2].

An assessment of the environmental variables influencing the distribution of *C. barometz* showed that precipitation levels during the driest quarter, total annual precipitation, the minimum temperature recorded in the coldest month, and the annual temperature range emerged as the dominant climatic constraints. Together these four factors account for approximately 94 percent of the explanatory power in the model, underscoring their central role in shaping where the species can persist. This result provides a clear measure of how strongly this species responds to both water availability and low temperature conditions. For healthy growth, the area needs at least 96.84 mm of rain during the three driest months of the year, while total yearly rainfall should ideally fall between 3834.10 and 4209.60 mm. In addition, temperatures during the coldest month must remain above 8.79 °C to support the species. These climatic requirements highlight the importance of stable moisture and moderate winter temperatures in shaping where this plant can successfully establish itself. From an ecophysiological perspective, these thresholds likely represent the fundamental moisture limits and thermal tolerance boundaries necessary for maintaining basic physiological functions. During dry seasons, insufficient precipitation may directly constrain critical life cycle stages including spore germination, gametophyte survival, and juvenile sporophyte establishment [62], which aligns closely with the well-documented water dependency characterizing pteridophyte biology. Previous research has substantiated that the natural habitats of *C*. *barometz* maintain an average annual temperature of approximately 17.9 °C, remarkably high relative humidity averaging 91.7%, and acidic soil conditions, collectively providing essential requirements for spore germination and gametophyte development [63]. Furthermore, cultivation observations indicate optimal growth temperatures ranging from 10 to 16 °C during nighttime and 21 to 26 °C during daytime, accompanied by substantial atmospheric moisture demands with relative humidity around 80% [64]. Such empirical evidence further corroborates the ecological validity of the minimum cold month temperature threshold (≥8.79 °C) identified in this study, as well as the species’ fundamental dependence on stable moisture conditions, thereby reinforcing the physiological plausibility of our modeled environmental constraints. It should be noted that variable importance estimates can vary among different species distribution models, i.e., variable importance is model-dependent. In this study, the MaxEnt model identified precipitation of the driest quarter (Bio17) as the most important contributor (52.6%), whereas the multi-model ensemble (RF, GAM, GBM, etc.) indicated that the mean temperature of the driest quarter (Bio9) had the highest average importance (0.473). This discrepancy arises from the inherent mechanisms of each algorithm: MaxEnt is based on the maximum entropy principle and is sensitive to marginal responses, while tree-based models (RF, GBM) and generalized additive models (GAM) are more adept at capturing non-linear interactions. To mitigate uncertainties from any single model, we adopted a multi-model ensemble strategy that integrates predictions from eight algorithms, thereby providing more robust estimates of suitable habitats. Therefore, when interpreting variable importance, one should consider consensus across multiple models rather than relying on a single algorithm.

At the level of model comparison, the implementation of a multi-model ensemble strategy markedly enhanced the stability and reliability of our predictions. The ensemble model yielded superior performance metrics, including AUC (0.964), TSS (0.842), and Kappa (0.794), which generally outperformed those of most individual models, thereby effectively mitigating the uncertainties stemming from algorithmic variability. Nevertheless, it is worth noting that the individual models displayed certain discrepancies in their responses to future climatic conditions. For instance, GAM and GBM tended to project an expansion of suitable habitats, whereas MaxEnt and RF exhibited more conservative trends (Figure 7, Supplementary Table S7). Such differences likely arise from the distinct mechanisms by which these algorithms handle nonlinear relationships and interaction effects, further underscoring the necessity of employing an ensemble modeling approach when evaluating multiple scenarios and time horizons [65,66,67]. Such discrepancies were especially evident under the high-emission SSP585 pathway, underscoring the limitations and potential risks of depending on any single modeling approach when projecting species responses under severe climate futures.

Simulations under future climate scenarios revealed a strong dependency of the distributional patterns of *C. barometz* on the trajectory of greenhouse gas emissions. Under the low-emission SSP126 scenario, the total area of suitable habitat remained largely unchanged or even showed modest growth, indicating that even moderate efforts to reduce emissions may help support population stability. By contrast, the high-emission SSP585 pathway led to notable shrinkage and increased fragmentation of suitable habitats, with the most severe impacts anticipated by the 2090s. Notably, RF and GAM predicted reductions in total suitable area of 31.76% and 7.68%, respectively, with highly suitable areas drastically diminishing to isolated patches in regions such as Hainan and Taiwan. This response pattern implies that sustained intensification of the greenhouse effect may exceed the species’ climatic tolerance thresholds, thereby posing a substantial threat to its long-term persistence.

Analysis of centroid shifts offered additional perspective on how *C. barometz* may adjust its spatial distribution in response to rising temperatures. According to the ensemble model projections, the current centroid of suitable habitat lies at the intersection of Yunnan, Guizhou, and Guangxi, specifically at 108.46° E and 26.99° N (Figure 8a, Supplementary Table S8). Looking toward the late century, distinct movement patterns emerge under different emissions scenarios. By the 2090s, the centroid is projected to shift markedly southwestward under the SSP245, SSP370, and SSP585 pathways, with displacement distances reaching 331.3 km, 335.1 km, and 180.2 km, respectively. These shifts converge toward mid to high elevation areas, particularly in southeastern Yunnan, southern Guizhou, and western Guangxi, suggesting these regions may serve as future climatic refugia for the species. Such a pattern of movement closely resembles the adaptive responses documented in other plant species, including *Cupressus gigantea* and *Lamiophlomis rotata*, both of which have been shown to shift toward higher elevations or latitudes under warming conditions [68,69]. This consistency across taxa points to a broader ecological pattern in how species adjust their distributions in response to climate change. In light of these findings, the eastern Yunnan-Guizhou Plateau together with its surrounding areas emerges as a potentially important climatic refuge for *C. barometz* in the decades ahead.

When contextualizing our national-scale analysis with the investigation by Feng et al. [20], which concentrated specifically on the Guangxi region, valuable complementary insights emerge. Their study employed an integrated approach combining MaxEnt with the PLUS model to explore the regional habitat dynamics of *C. barometz* under the synergistic impacts of climate and land use change. Their investigation revealed that by the 2040s, suitable areas for the species within Guangxi were projected to contract under the SSP126 and SSP370 scenarios, whereas a slight expansion was anticipated under the SSP585 pathway. At the same time, the centroid of suitable habitat within the province shifted markedly toward higher elevations in the northeast. This comparison with our work highlights a multi-faceted complementarity. Firstly, concerning spatial scale, our research provides a comprehensive national overview, pinpointing core distribution zones in southeastern Yunnan, southern Guizhou, and western Guangxi. In contrast, the focused analysis by Feng et al. [20] on the primary production area of Guangxi delivers essential insights for fine-scale, regional management, thereby creating a hierarchical understanding. Secondly, regarding methodological approach, our study leverages a robust multi-model ensemble strategy, encompassing nine algorithms and a parameter-optimized MaxEnt, to systematically evaluate climatic response patterns across the entire country, which effectively mitigates the uncertainties inherent in single-model predictions. While Feng et al. [20] advanced the analysis by incorporating land use dynamics, their methodological framework may present certain limitations in algorithmic robustness compared to our ensemble approach. Furthermore, in terms of identifying key environmental influences, our results highlight precipitation during the driest quarter, annual precipitation, minimum temperature of the coldest month, and temperature annual range as the primary climatic factors shaping habitat suitability, together explaining 94 percent of the model’s predictive power. By contrast, Feng and colleagues [20] emphasize the importance of elevation and precipitation in the warmest quarter as dominant drivers in their regional analysis. This divergence may be attributable to differences in study scale and regional environmental heterogeneity, as local topographical and climatic nuances within Guangxi could amplify the importance of factors like elevation. It is important to note that the southwestward migration trend at the national scale identified in our study is not inconsistent with the northeastward shift towards higher elevations within Guangxi reported by Feng et al. [20]. Rather, these findings elegantly illustrate a hierarchical response to climate change operating at different spatial levels: a broad-scale contraction towards the southwestern interior of the country, alongside a regional-scale vertical migration towards higher altitudes within a specific province. This scale-dependent pattern of migration provides a robust scientific rationale for formulating multi-level conservation strategies that operate coherently from the national down to the local level. Collectively, these two studies, by corroborating and complementing each other’s findings, significantly deepen our comprehensive understanding of how *C. barometz* responds to the multifaceted challenges of climate change.

A critical consideration is that while our models project the emergence of new suitable habitats in certain regions under future scenarios, the actual capacity of *C. barometz* populations to successfully migrate and establish in these areas remains subject to multiple constraints. Given that this species primarily reproduces via spores, its natural dispersal capabilities are inherently limited. This ecological constraint is compounded by the persistent pressures of habitat fragmentation and historical anthropogenic harvesting on its wild populations, suggesting that the actual rate of species migration may lag considerably behind the velocity at which climatic suitability shifts [62,70]. Consequently, proactive conservation interventions will be paramount for mitigating future climate risks. These should strategically prioritize the establishment of artificial propagation programs and ex situ conservation sites within the prospective high-suitability zones identified in regions like Guangxi and Yunnan. Furthermore, the development of ecological corridors designed to enhance landscape connectivity will be essential for facilitating natural dispersal and gene flow [71].

Furthermore, the species distribution models used in this study implicitly assume that *C. barometz* is in equilibrium with current climate conditions [46]. However, wild populations of this species have been subject to long-term harvesting pressure, with sustained increases in market demand. Zhu et al. [72] analyzed the use of antirheumatic TCM decoction pieces in a tertiary hospital from 2010 to 2014 and found that the annual consumption of *C. barometz* increased from 1548 kg to 2562 kg, representing a 65.5% increase over five years, reflecting strong clinical demand for this medicinal material. This continued market pressure exacerbates over-harvesting of wild resources, leading to significant population declines or even local extinctions within suitable areas. Additionally, the species reproduces primarily by spores with limited natural dispersal capacity, and habitat fragmentation further restricts its colonization of potentially suitable habitats. These factors together suggest that the current distribution of *C. barometz* may underestimate its climatic potential—i.e., the species exhibits incomplete range filling [45]. Consequently, our predictions may be conservative in areas with high anthropogenic pressure, underestimating climatic suitability under natural conditions. Conversely, for newly predicted suitable areas under future scenarios (e.g., southeastern Yunnan, western Guangxi), actual colonization success remains highly uncertain due to limited dispersal ability and anthropogenic barriers, and these predictions may be overly optimistic. It should be noted that the occurrence data used in this study were primarily derived from historical specimen records (CVH, NSII, GBIF), without distinguishing between wild and cultivated individuals or excluding areas where populations have been extirpated due to historical over-harvesting. In such areas, environmental conditions may remain suitable, but the species is locally extinct due to human activities, resulting in “false absences” in the data. This bias may lead to underestimation of climatic suitability in areas with high anthropogenic pressure [16]. Future studies should incorporate anthropogenic pressure variables (e.g., harvesting intensity, distance to roads, population density, land-use change) as predictors or use bias files to correct occurrence data. We recommend that future studies integrate population dynamics models, dispersal capacity assessments, and anthropogenic disturbance scenarios to more accurately predict the species’ response to climate change.

It should be noted that the occurrence data used in this study span from 1869 to 2025, covering more than 150 years. Species distribution models typically assume equilibrium between species and their environment [73], and such a long temporal span may introduce non-equilibrium changes due to climate change and anthropogenic pressure. Although *C. barometz* is a long-lived perennial fern with a relatively stable historical distribution, and we retained all records to capture the full environmental gradient, temporal mismatch may still cause some bias. To assess this potential effect, we conducted a sensitivity analysis using only the last 30 years of data (1995–2025). The results showed that the dominant environmental drivers (Bio17, Bio12, Bio6, Bio7), their suitable thresholds, and the direction of centroid shifts remained highly consistent with those obtained from the full dataset, indicating that temporal mismatch has limited impact on our core conclusions. Nevertheless, we recommend that future studies preferentially use recent data or adopt time-explicit modeling approaches when feasible.

Beyond the temporal span, the source of occurrence data also deserves attention. The presence records used in this study were drawn from herbarium specimens, which may include cultivated individuals. Cultivated records can introduce bias: for instance, through artificial irrigation or shading, cultivated plants may persist in areas that are naturally unsuitable, potentially leading the model to overestimate water requirements or underestimate temperature tolerance [43]. Because specimen records lack source information, we could not effectively distinguish between wild and cultivated sources at the data level. To mitigate potential impacts, we applied robust methods including spatial filtering and multi-model ensemble. Notably, traditional cultivation of *C. barometz* generally occurs within its natural range, and our core conclusions (e.g., precipitation of the driest quarter and minimum temperature of the coldest month as dominant drivers) are highly consistent with the species’ ecological traits, suggesting that the distortion from cultivated records is likely limited. Nevertheless, future studies should explicitly record source information or use independent datasets of wild occurrences for validation.

One important consideration is that pseudo-absence generation strategies can potentially affect species distribution model performance. In this study, we adopted random pseudo-absence generation across the entire study area (administrative boundary of China), which is simple, reproducible, and computationally efficient. Wisz and Guisan [74] demonstrated through a virtual species experiment that models built with randomly selected pseudo-absences, despite having lower fit, achieved high predictive power (AUC) and outperformed two-step pseudo-absence selection strategies, concluding that random pseudo-absences are a reasonable alternative when true absences are unavailable. Nevertheless, we acknowledge that random sampling may ignore environmental structure, and future studies may explore environmentally stratified sampling or target-group background sampling [42]. Our core conclusions, validated through multi-model ensemble cross-validation, are robust to pseudo-absence sampling choices.

Furthermore, species distribution data often exhibit spatial autocorrelation, and conventional random cross-validation may underestimate prediction error [54]. To test the sensitivity of our models to spatial structure, we conducted spatial block cross-validation. The results showed that the spatial validation AUC values were almost identical to those from random validation (difference < 0.002), and all core conclusions (dominant environmental drivers, spatial patterns of suitable habitats, centroid shift trends) remained unchanged. This confirms that our models are robust to spatial autocorrelation, and random cross-validation did not lead to significant overestimation of performance. Nevertheless, we recommend that future studies preferentially use spatial block cross-validation [55] when conditions permit, to obtain more conservative and reliable performance estimates.

Regarding sampling bias treatment, this study did not implement standard methods such as bias files (target-group background) or accessibility masks [42]. This was primarily because reliable sampling effort data (e.g., collector preferences, road networks) were unavailable for the herbarium records of *C. barometz*. As alternatives, we applied spatial filtering (5 km thinning) and a multi-model ensemble to mitigate bias. Nevertheless, we recommend that future studies, when feasible, use target-group backgrounds or bias files to directly correct sampling bias and explicitly define the accessible area M within the BAM framework.

It should be noted that the interpretation of SSP scenarios in this study should avoid simplistic linearization. Although SSP585 represents a high-forcing pathway and SSP126 a low-emission sustainability pathway, the recent literature [75] indicates that the near-term emissions trajectory of SSP585 may be overestimated and should not be treated as a “business-as-usual” certainty. Therefore, comparisons between different SSP scenarios should be interpreted cautiously, taking model variability into account. In this study, the multi-model ensemble (RF, GAM, GBM, MaxEnt) shows some variability in projected suitable area changes and centroid shift distances under each SSP scenario (see Results Section 3.4 and Section 3.5 and Supplementary Tables S7 and S8). These uncertainties reflect different algorithmic responses to climatic variables. Despite this variability, all models consistently indicate that high-emission scenarios lead to contraction and fragmentation of suitable habitats, whereas low-emission scenarios support relative stability. Thus, the core conclusions of this study are robust across scenarios, but quantitative comparisons should consider the reported uncertainty ranges.

It should be noted that this study used only one GCM (BCC-CSM2-MR) to generate future climate scenarios. Although this model performs well in simulating temperature and precipitation over China, the prediction uncertainty arising from differences in dynamical frameworks, parameterization schemes, and resolutions among different GCMs cannot be fully captured [76]. Previous studies have shown that even multi-model ensemble means may fail to reproduce certain regional climate features [77], so projections from a single GCM must be interpreted with caution. Therefore, our predictions should be regarded as scenario-based trend estimates under BCC-CSM2-MR rather than deterministic conclusions. Future studies should incorporate multiple GCMs (e.g., CESM2, MPI-ESM1-2-HR from CMIP6) to quantify inter-model uncertainty and provide more reliable projections of suitable habitats.

It is also important to acknowledge that despite our rigorous approach to model construction and environmental variable screening, this study inherently possesses certain limitations that warrant consideration. Firstly, our framework does not fully incorporate the potentially modifying influences of future land use change, soil dynamics, or biotic interactions such as interspecific competition, all of which can significantly shape realized habitat suitability [78]. Secondly, the capacity of current models to accurately simulate species responses to extreme climatic events, including prolonged droughts or intense precipitation episodes, remains an area requiring further refinement [79]. Moreover, our predictions are based on a static equilibrium assumption regarding the relationship between the species and its environment, and therefore do not account for potential physiological adaptations or phenotypic plasticity that *C. barometz* might exhibit in response to changing climatic conditions. Addressing these gaps in future research could involve integrating multi-source data and incorporating dynamic vegetation models alongside modules that simulate anthropogenic activities [80,81]. When combined with population genomics and remote sensing technologies, this integrated approach would support a more comprehensive risk assessment across multiple spatial scales [82,83]. Such methodological advancements stand to offer a more accurate scientific basis for guiding the conservation and sustainable use of *C*. *barometz* germplasm resources.

## 5. Conclusions

This investigation employed a multi-model ensemble framework to characterize the prospective suitable habitats of *C. barometz* under both contemporary and future climatic conditions, while simultaneously pinpointing the principal environmental determinants shaping its distributional range. Precipitation during the driest quarter, annual precipitation, minimum temperature of the coldest month, and temperature annual range were identified as the predominant climatic regulators, jointly accounting for 94% of the explained variation in the models. Favorable conditions for the species require dry-season precipitation exceeding 96.84 mm, annual precipitation within the range of 3834.10 to 4209.60 mm, coldest month minimum temperatures above 8.79 °C, and an optimal temperature annual range approximating 20.47 °C. These quantitative thresholds clearly delineate the species’ inherent ecological affinity for environments characterized by abundant moisture and moderate winter temperatures.

Currently, suitable habitats are largely restricted to the hilly and mountainous landscapes of southern Yunnan, Guangxi, Guangdong, Fujian, Hainan, and Taiwan, encompassing roughly 1.707 million square kilometers or 17.78% of China’s total land area. The observed fragmentation across these zones points to pronounced niche specificity, indicating that the species favors discrete microhabitats rather than uniformly occupying all climatically amenable regions. Future climate projections reveal substantial shifts in distribution patterns closely tied to emission trajectories. Under the low-emission SSP126 pathway, total suitable area contracts modestly by approximately 6% by mid-century before stabilizing toward the 2090s, with enhanced suitability emerging in certain mid- to high-altitude zones. In sharp contrast, the high-emission SSP585 scenario suggests pronounced habitat contraction and fragmentation, with total suitable area diminishing by as much as 0.07% to 31.76% by late century and highly suitable zones retreating to isolated refugia in Hainan and Taiwan, implying that escalating climatic pressure may exceed the species’ physiological thresholds.

Centroid shift dynamics further illuminate the spatial adaptation strategy of *C. barometz*. The current distribution centroid lies at the tri-provincial convergence of Yunnan, Guizhou, and Guangxi. By the 2050s, it will shift southward by 120 to 154 km under all examined scenarios. By the 2090s, trajectories diverge notably: continued southeastward progression of approximately 71 km under the low-emission scenario, compared to pronounced southwestward displacement of 180 to 335 km under moderate- to high-emission scenarios, ultimately converging in southeastern Yunnan, southern Guizhou, and western Guangxi. This observed contraction toward higher elevations within southwestern interior regions aligns with broader expectations of climate-driven migration toward potential refugia.

From a conservation perspective, these findings underscore the urgency of establishing germplasm conservation facilities and ex situ protection sites within the identified future core areas. Such efforts should be complemented by targeted habitat restoration and active management of anthropogenic disturbances across existing distribution ranges. Integrating advanced propagation techniques with strategically designed ecological corridors could bolster population dispersal capacity and adaptive potential, thereby mitigating projected climate-induced pressures. It is important to acknowledge certain limitations inherent in this study, including potential spatial sampling biases associated with herbarium-derived occurrence records, intrinsic uncertainties embedded within climate projections, and the omission of land use dynamics and biotic interactions from the modeling framework. Future research efforts would benefit from incorporating dynamic vegetation models and genomic approaches to enable more comprehensive, multi-scale risk assessments.

## Figures and Tables

**Table 1:** All environmental variables.

Variables	Name	Unit
Bio1	Annual mean temperature	°C
Bio2	Mean diurnal temperature range	°C
Bio3	Isothermality	/
Bio4	Temperature seasonality	/
Bio5	Maximum temperature of the warmest month	°C
Bio6	Minimum temperature of the coldest month	°C
Bio7	Temperature annual range	°C
Bio8	Mean temperature of the wettest quarter	°C
Bio9	Mean temperature of the driest quarter	°C
Bio10	Mean temperature of the warmest quarter	°C
Bio11	Mean temperature of the coldest quarter	°C
Bio12	Annual precipitation	mm
Bio13	Precipitation of the wettest month	mm
Bio14	Precipitation of the driest month	mm
Bio15	Precipitation seasonality	/
Bio16	Precipitation of the wettest quarter	mm
Bio17	Precipitation of the driest quarter	mm
Bio18	Precipitation of the warmest quarter	mm
Bio19	Precipitation of the coldest quarter	mm
Awc_class	Soil available water content	%
Slope	Slope	◦
Elev	Elevation	m
Aspect	Aspect	/
T_ph_h2o	Topsoil pH	−log(H+)
S_ph_h2o	Subsoil pH	−log(H+)
T_oc	Topsoil organic carbon content	% weight
S_oc	Subsoil organic carbon content	% weight
T_clay	Topsoil clay content	% weight
S_clay	Subsoil clay content	% weight
T_sand	Topsoil sand content	% weight
S_sand	Subsoil sand content	% weight
T_silt	Topsoil silt content	% weight
S_silt	Subsoil silt content	% weight
T_ece	Topsoil electrical conductivity	dS/m
S_ece	Subsoil electrical conductivity	dS/m
T_caco3	Topsoil carbonate or lime content	% weight
S_caco3	Subsoil carbonate or lime content	% weight

**Table 2:** Performance metrics (AUC, TSS, and Kappa) of the eight species distribution models evaluated for predicting the potential geographic distribution of *C. barometz*.

Indicator	Algorithm							
	RF	GAM	GBM	MARS	CTA	GLM	ANN	SRE
AUC	0.9381	0.9309	0.9363	0.9247	0.8863	0.8339	0.8334	0.6959
TSS	0.6539	0.7563	0.7509	0.7326	0.7650	0.6677	0.6668	0.3920
Kappa	0.6043	0.6157	0.6276	0.5904	0.5765	0.5440	0.4734	0.4526

**Table 3:** Importance of key environmental factors affecting the distribution of *C. barometz* in China, as derived from the optimized MaxEnt model.

Variable	Name	Percent Contribution (%)	Permutation Importance (%)
Bio17	Precipitation of the driest quarter	52.6	10.4
Bio12	Annual precipitation	24.9	9.1
Bio6	Minimum temperature of the coldest month	10.2	22.3
Bio7	Temperature annual range	6.3	45.9
S_oc	Subsoil organic carbon content	2.1	2.9
Slope	Slope	1.9	2.6
T_silt	Topsoil silt content	0.5	0.6
Bio5	Maximum temperature of the warmest month	0.5	2.3
Bio15	Precipitation seasonality	0.5	2
T_clay	Topsoil clay content	0.2	1.1
Awc_class	Soil available water content	0.2	0.5
Aspect	Aspect	0.1	0.2

**Table 4:** The suitable range for the dominant environmental factors.

Variable	Suitable Range	Adaptive Threshold
Bio17	3.25~640.20 mm	96.84 mm
Bio12	74.58~4209.60 mm	3834.10~4209.60 mm
Bio6	−35.41~22.35 °C	8.79 °C
Bio7	16.25~27.92 °C	20.47 °C
S_oc	0.03~42.30%	0.49%
Slope	89.60~90.03°	89.92°

Note: The “suitable range” is the interval where the predicted probability >0.5; extreme values are mathematical boundaries. The “adaptive threshold” is the ecologically critical indicator.

## Data Availability

The original contributions presented in this study are included in the article/Supplementary Materials. Further inquiries can be directed to the corresponding authors.

## Supplementary Materials

The following supporting information can be downloaded at: https://www.mdpi.com/article/10.3390/biology15090692/s1, Figure S1: Accuracy verification results for eight single-model algorithms. Figure S2: Correlation heatmap of environmental factors. Figure S3: Potential suitable habitats of *C. barometz* predicted by different models under the SSP126 scenario for the 2090s. Figure S4: Potential suitable habitats of *C. barometz* predicted by different models under the SSP245 scenario for the 2050s. Figure S5: Potential suitable habitats of *C. barometz* predicted by different models under the SSP245 scenario for the 2090s. Figure S6: Potential suitable habitats of *C. barometz* predicted by different models under the SSP370 scenario for the 2050s. Figure S7: Potential suitable habitats of *C. barometz* predicted by different models under the SSP370 scenario for the 2090s. Figure S8: Potential suitable habitats of *C. barometz* predicted by different models under the SSP585 scenario for the 2050s. Figure S9: MaxEnt model predictions under current climate conditions. Figure S10: Potential distribution of *C. barometz* under different future climate scenarios as modelled by MaxEnt using 128 temporally filtered occurrence points (1995–2025). Figure S11: Potential distribution of *C. barometz* under future climate scenarios as modelled by MaxEnt using the fixed threshold (10th percentile training presence Cloglog = 0.3927). Figure S12: Centroid migration trajectories of suitable habitats for *C. barometz*. Table S1: Variance Inflation Factor (VIF) values for the retained environmental variables. Table S2: Average variable importance across eight algorithms (RF, GBM, MARS, GAM, GLM, CTA, ANN, SRE) based on 10 repeated runs. Table S3: Parameter settings for all component models in the ensemble. Table S4: Continuous Boyce Index (CBI) for the four models in the final ensemble. Table S5: Comparison of AUC values between random cross-validation and spatial block cross-validation for the four models in the final ensemble. Table S6: Comparison of three ensemble methods for predicting the potential distribution of *C. barometz*. Table S7: Suitable area of *C. barometz* under different future climate scenarios and periods (for MaxEnt, RF, GAM, GBM, and Ensemble Model). Table S8: Centroid coordinates and migration distances of suitable habitats for *C. barometz* under different climate scenarios and periods across five modeling approaches (MaxEnt, RF, GAM, GBM, and Ensemble Model). Table S9: Suitable area and centroid shifts for *C. barometz* based on 128 temporally filtered occurrence points (1995–2025) under different climate scenarios and periods (MaxEnt model). Table S10: Suitable area and centroid shifts for *C. barometz* based on the fixed threshold (10th percentile training presence Cloglog = 0.3927) under different climate scenarios and periods (MaxEnt model).
