# Supplementary material for: Prediction of Potential Habitat Distribution of Cibotium barometz (L.) J. Sm. Under Climate Change Based on a Multi-Model Ensemble Framework
Source: Biology (Basel). 2026 Apr 28;15(9):692. doi: 10.3390/biology15090692 (PMC13162567; doi:10.3390/biology15090692)
Supplement: Supplementary file 1 [file biology-15-00692-s001.zip › biology-4243077-supplementary.docx]

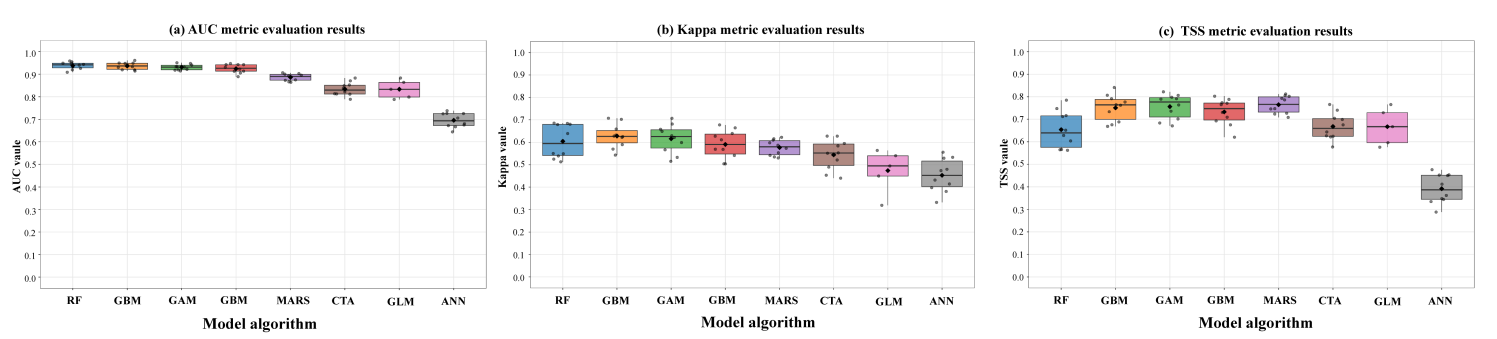


**Figure S1.** Accuracy verification results for eight single-model algorithms. (a) AUC metric evaluation results; (b) Kappa metric evaluation results; (c) TSS metric evaluation results.


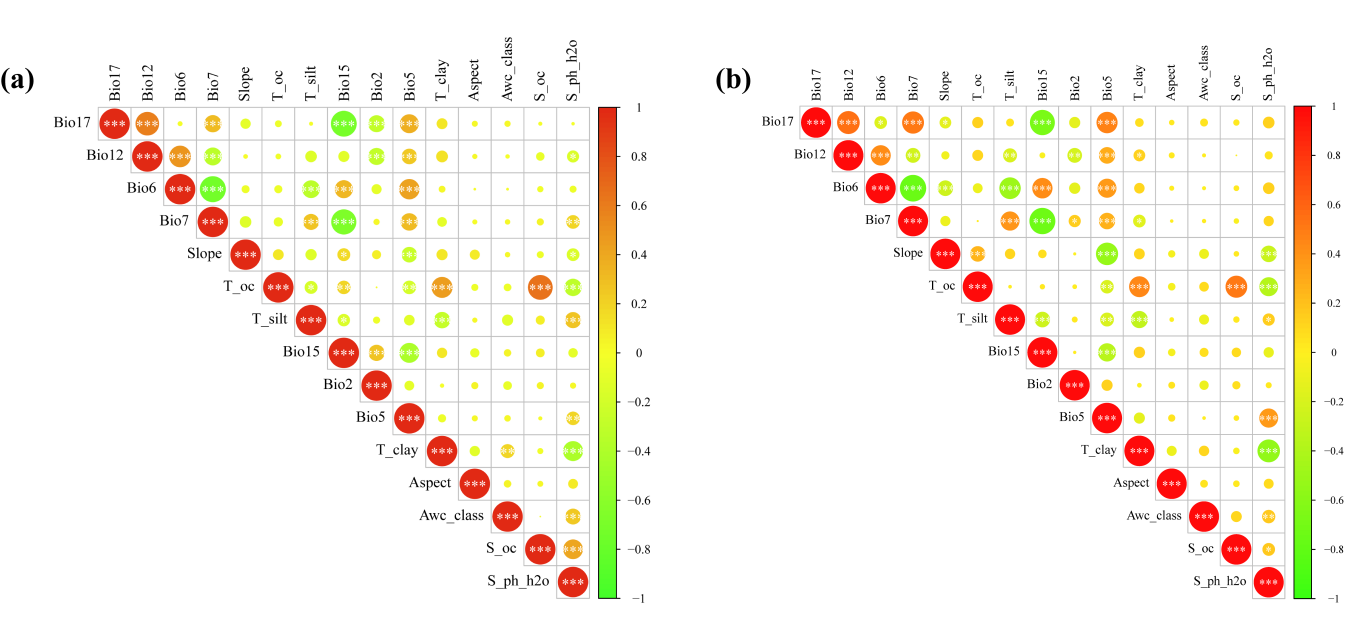


**Figure S2.** Correlation Heatmap of Environmental Factors: (a) Pearson correlation coefficients; (b) Spearman’s rank correlation coefficients.


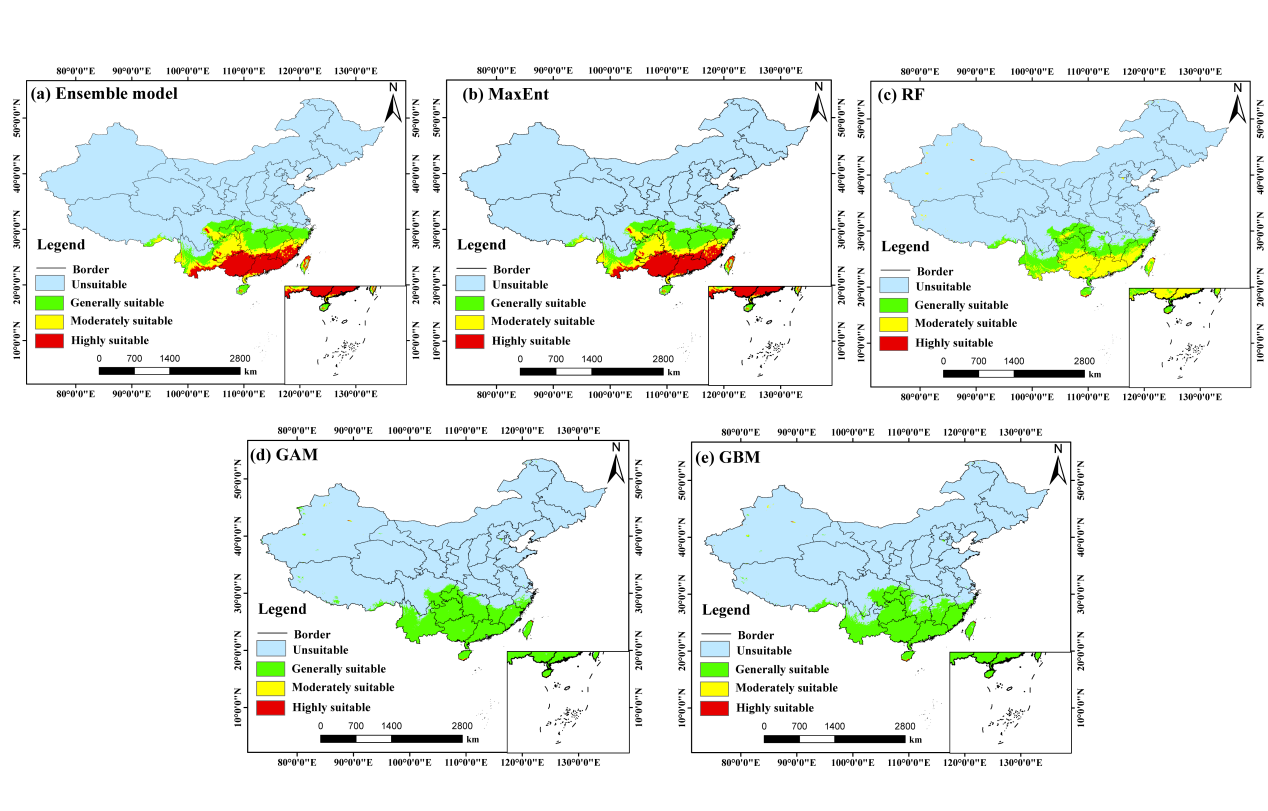


**Figure S3.** Potential suitable habitats of *C. barometz* predicted by different models under the SSP126 scenario for the 2090s: (a) Ensemble model; (b) MaxEnt; (c) RF; (d) GAM; (e) GBM.


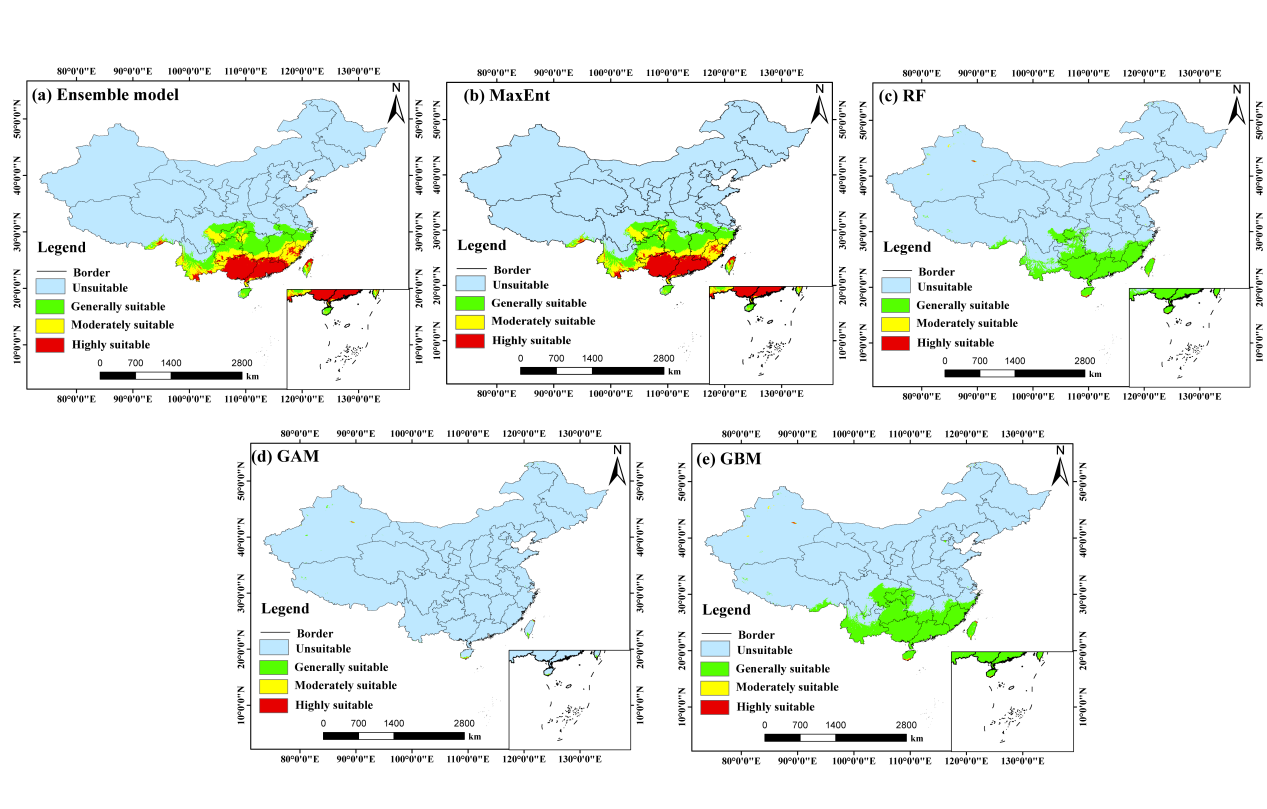


**Figure S4.** Potential suitable habitats of *C. barometz* predicted by different models under the SSP245 scenario for the 2050s: (a) Ensemble model; (b) MaxEnt; (c) RF; (d) GAM; (e) GBM.


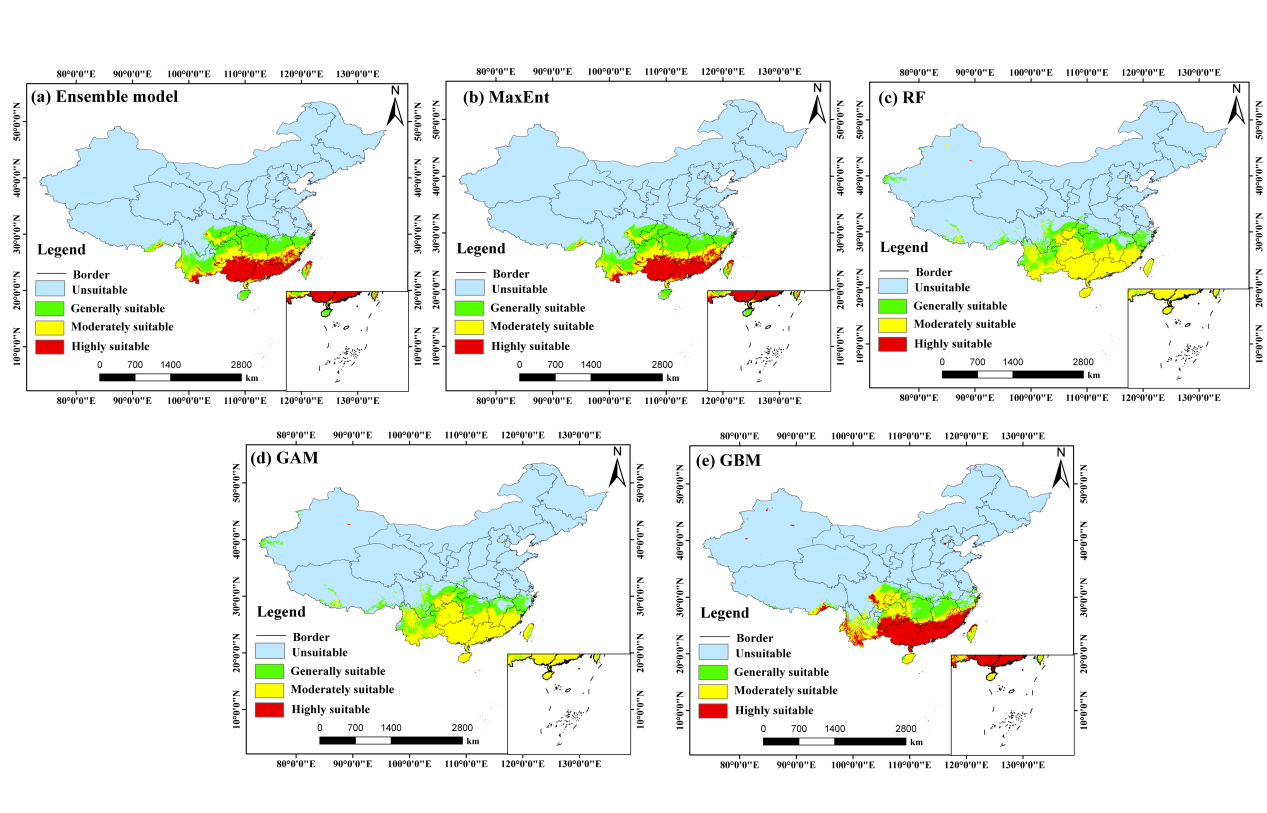


**Figure S5.** Potential suitable habitats of *C. barometz* predicted by different models under the SSP245 scenario for the 2090s: (a) Ensemble model; (b) MaxEnt; (c) RF; (d) GAM; (e) GBM.


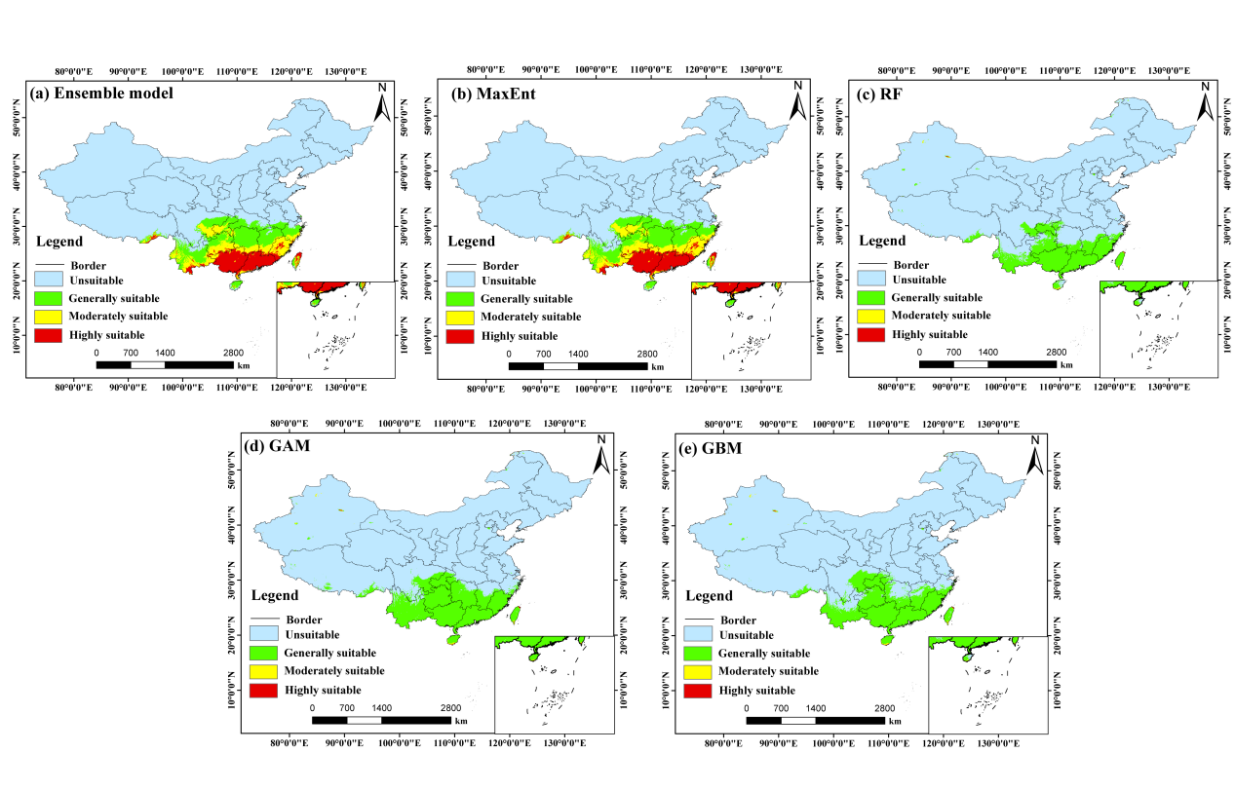


**Figure S6.** Potential suitable habitats of *C. barometz* predicted by different models under the SSP370 scenario for the 2050s: (a) Ensemble model; (b) MaxEnt; (c) RF; (d) GAM; (e) GBM.


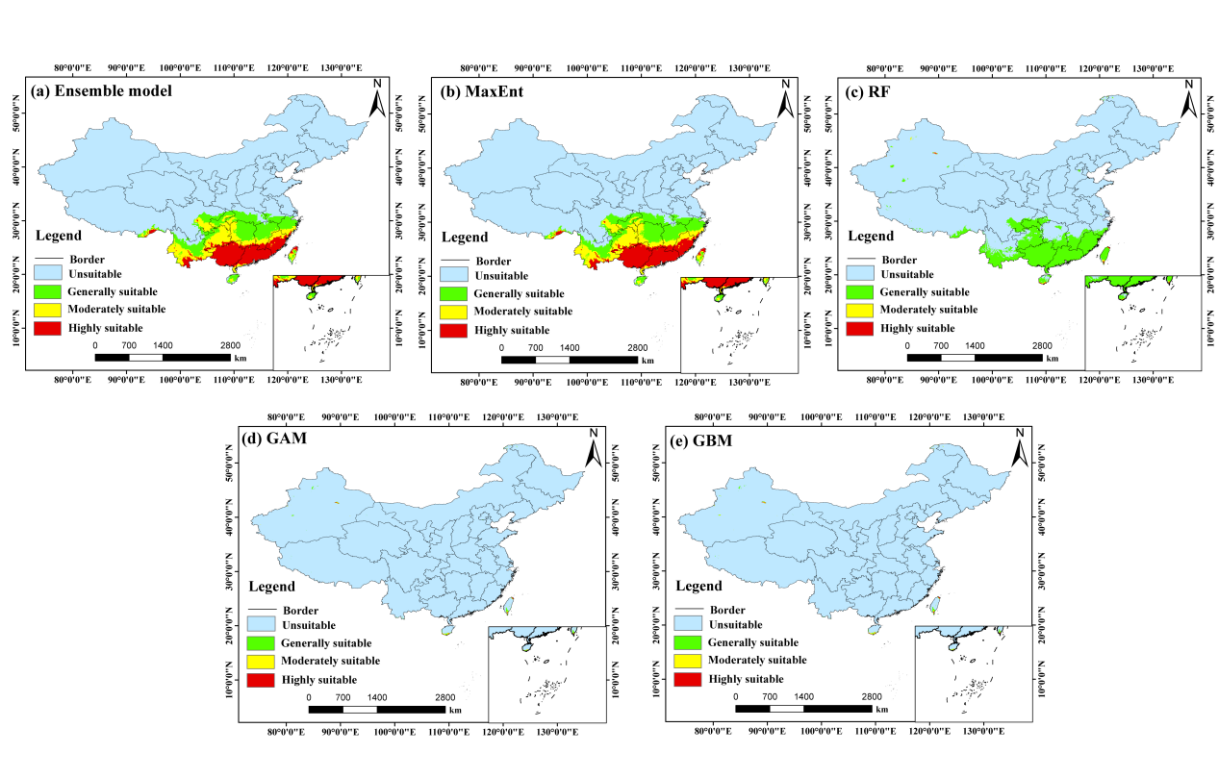


**Figure S7.** Potential suitable habitats of *C. barometz* predicted by different models under the SSP370 scenario for the 2090s: (a) Ensemble model; (b) MaxEnt; (c) RF; (d) GAM; (e) GBM.


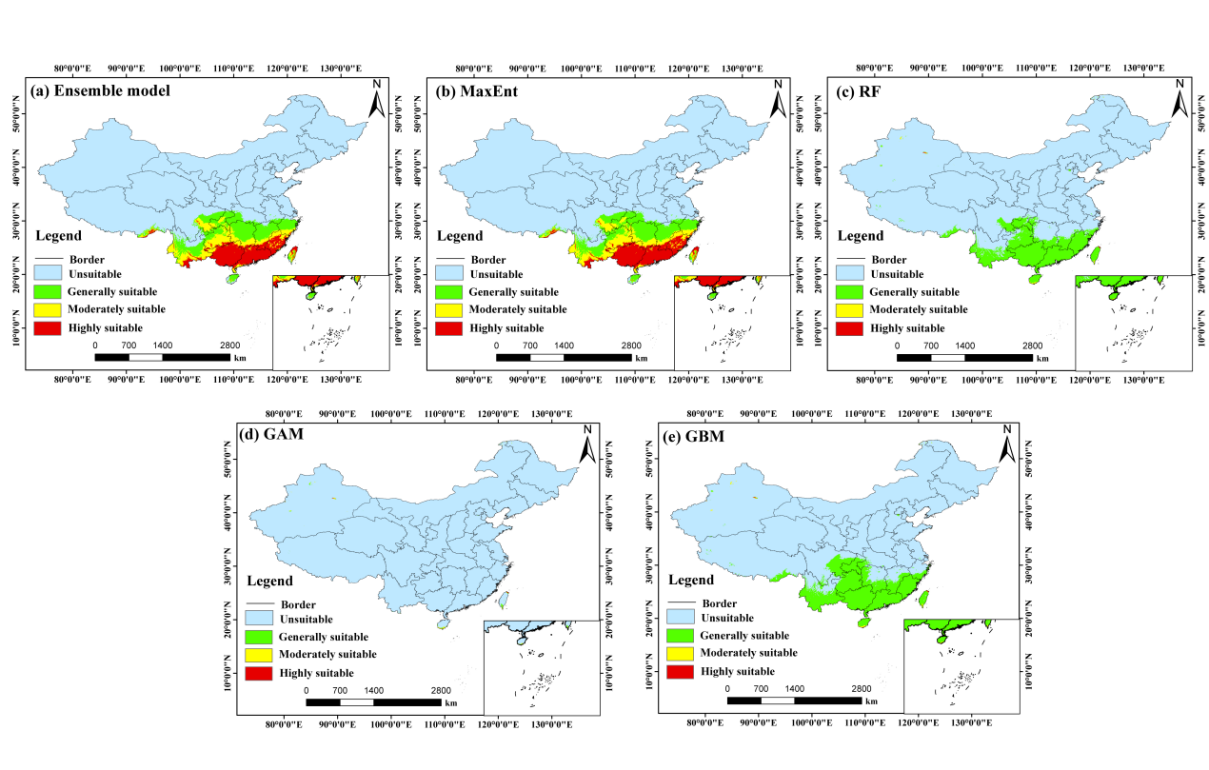


**Figure S8.** Potential suitable habitats of *C. barometz* predicted by different models under the SSP585 scenario for the 2050s: (a) Ensemble model; (b) MaxEnt; (c) RF; (d) GAM; (e) GBM.


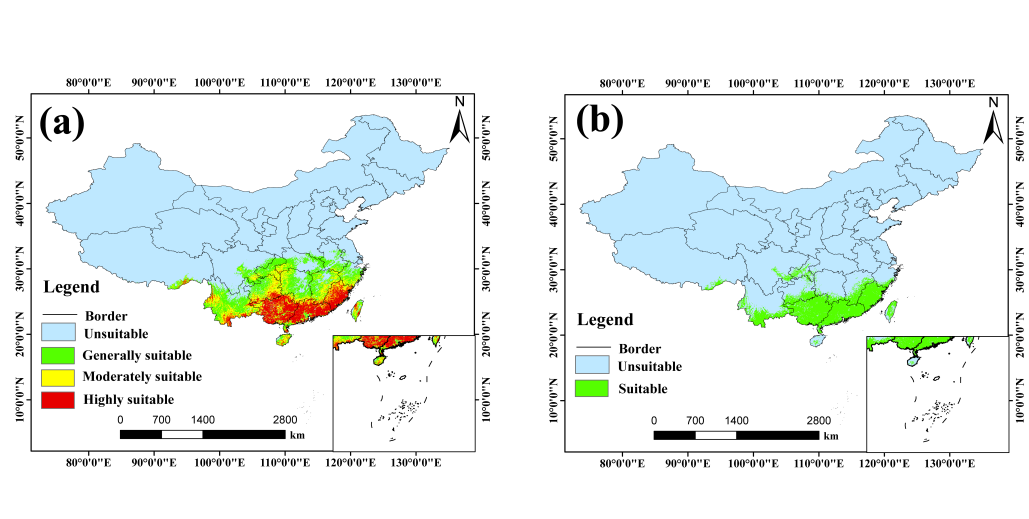


**Figure S9.** MaxEnt model predictions under current climate conditions: (a) using 128 temporally filtered occurrence points (1995–2025); (b) suitable area based on the fixed threshold (10th percentile training presence Cloglog = 0.3927).


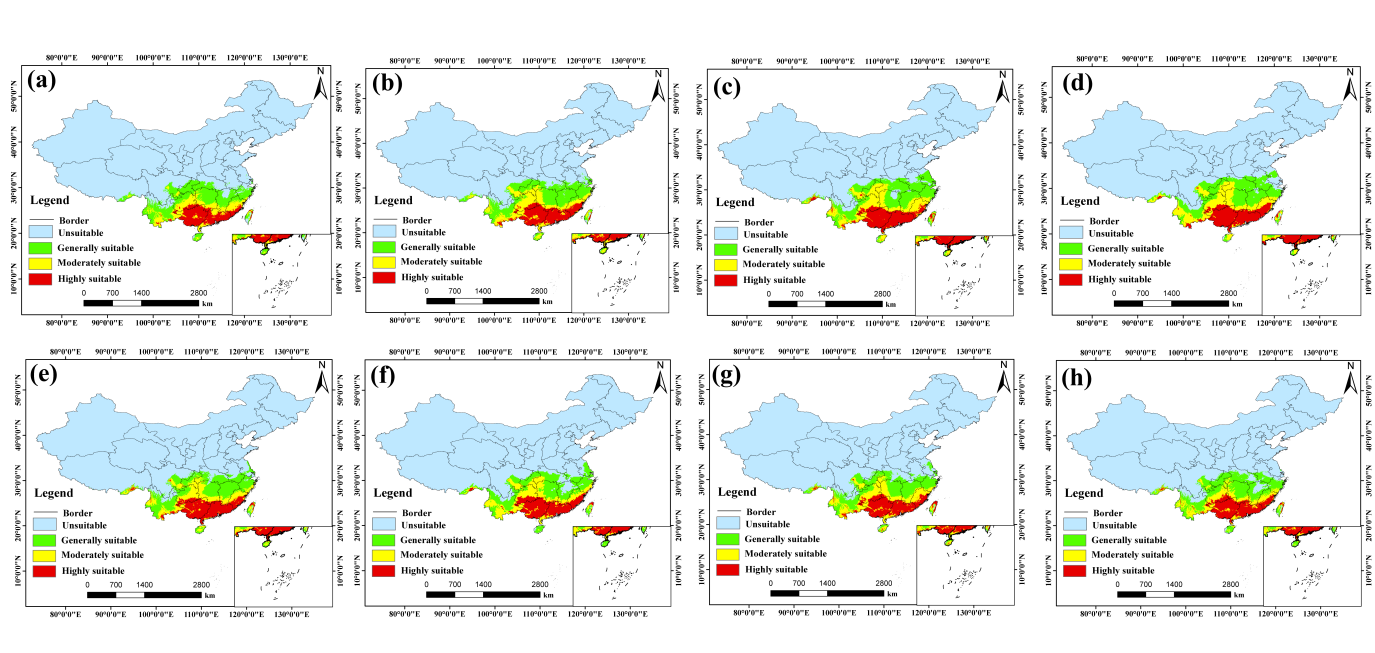


**Figure S10.** Potential distribution of *C. baromet*z under different future climate scenarios as modelled by MaxEnt using 128 temporally filtered occurrence points (1995–2025). The panels show: (a) 2041–2060, SSP126; (b) 2081–2100, SSP126; (c) 2041–2060, SSP245; (d) 2081–2100, SSP245; (e) 2041–2060, SSP370; (f) 2081–2100, SSP370; (g) 2041–2060, SSP585; (h) 2081–2100, SSP585.


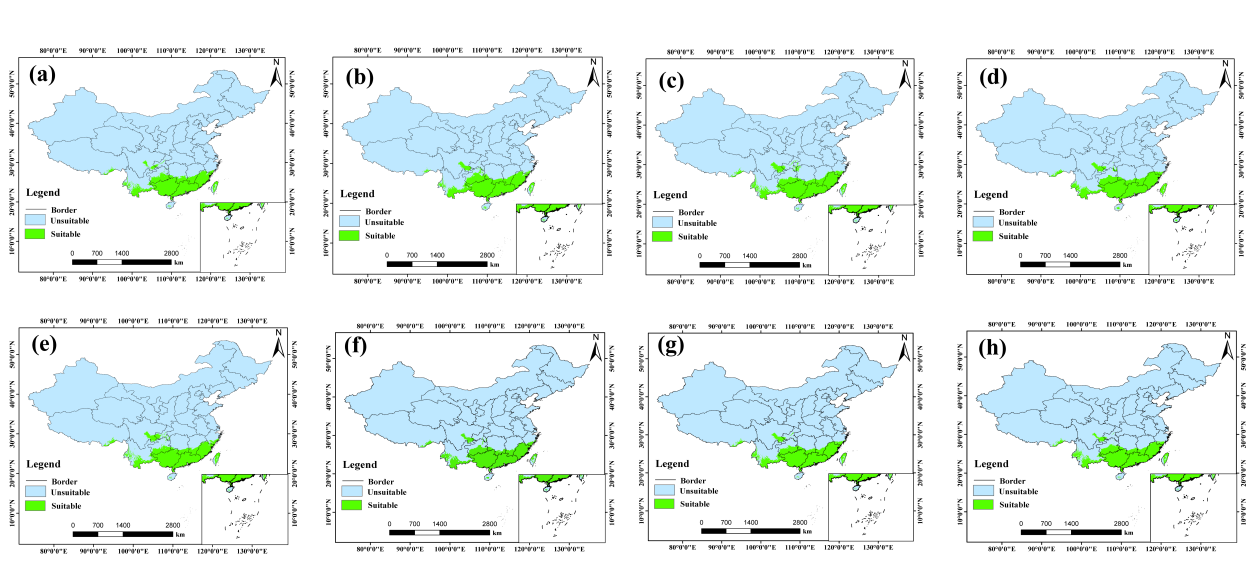


**Figure S11.** Potential distribution of *C. barometz* under future climate scenarios as modelled by MaxEnt using the fixed threshold (10th percentile training presence Cloglog = 0.3927). The panels show: (a) 2041–2060, SSP126; (b) 2081–2100, SSP126; (c) 2041–2060, SSP245; (d) 2081–2100, SSP245; (e) 2041–2060, SSP370; (f) 2081–2100, SSP370; (g) 2041–2060, SSP585; (h) 2081–2100, SSP585.

**Note:** The fixed threshold (0.3927) was derived as the mean 10th percentile training presence Cloglog threshold from 10‑fold cross‑validation of the optimized MaxEnt model under current climate (1970–2000). Suitable areas (green) indicate regions with predicted occurrence probability ≥ 0.3927; unsuitable areas (white) indicate probability < 0.3927.


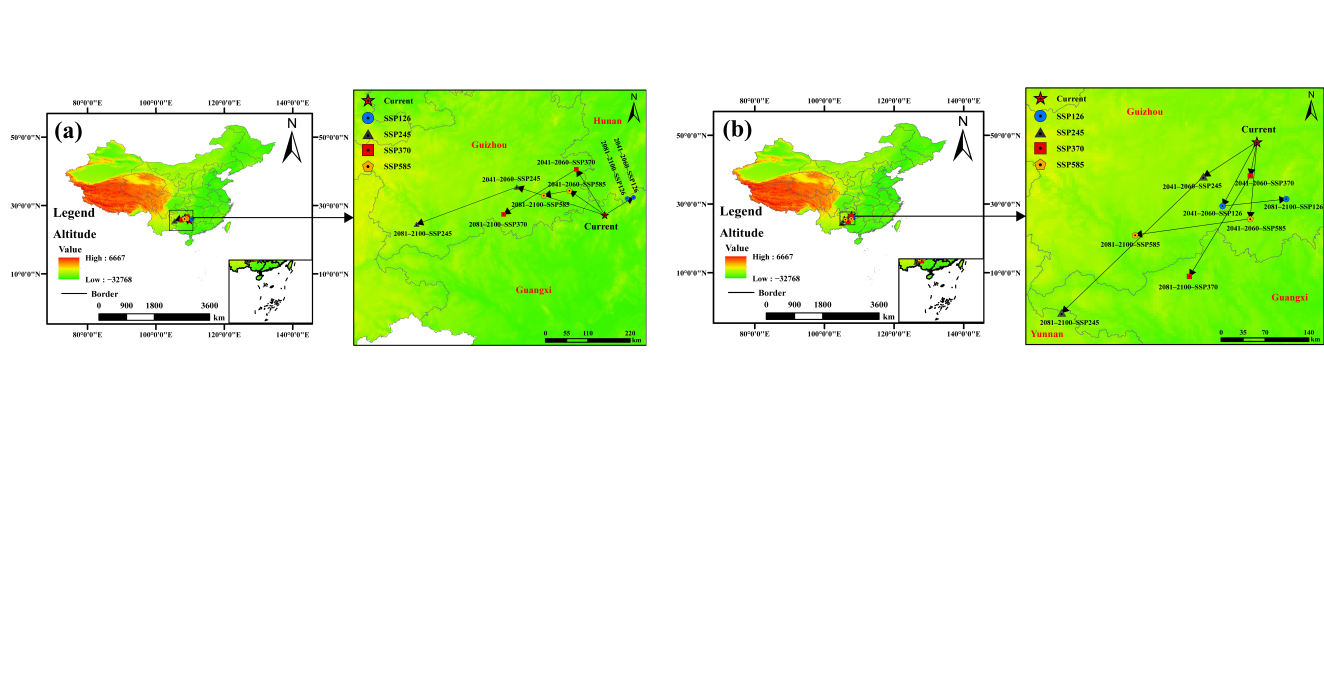


**Figure S12.** Centroid migration trajectories of suitable habitats for *C. barometz*: (a) based on 128 temporally filtered occurrence points (1995–2025); (b) suitable area and centroid shifts under different climate scenarios and periods using the fixed threshold (10th percentile training presence Cloglog = 0.3927) derived from the MaxEnt model.

**Table S1.** Variance Inflation Factor (VIF) values for the retained environmental variables (VIF < 5 indicates acceptable multicollinearity).

| **Variable** | **VIF** |
| --- | --- |
| Bio12 | 1.676 |
| Bio6 | 2.798 |
| Bio7 | 4.102 |
| S_oc | 1.031 |
| Slope | 1.123 |
| T_silt | 1.252 |
| Bio15 | 2.552 |
| T_clay | 1.185 |
| Awc_class | 1.063 |
| Aspect | 1.042 |

**Note:** Bio5 and Bio17 were excluded from this table due to perfect collinearity, but their ecological importance is addressed in the text.

**Table S2.** Average variable importance across eight algorithms (RF, GBM, MARS, GAM, GLM, CTA, ANN, SRE) based on 10 repeated runs. Only variables with consistent high importance are shown.

| **Variable** | **Mean Importance** | **SD** | **Rank** |
| --- | --- | --- | --- |
| Bio_9 (Mean Temp of Driest Quarter) | 0.473 | 0.361 | 1 |
| Bio_14 (Precip of Driest Month) | 0.218 | 0.327 | 2 |
| Bio_13 (Precip of Wettest Month) | 0.212 | 0.376 | 3 |
| Bio_15 (Precipitation Seasonality) | 0.178 | 0.333 | 4 |
| Bio_2 (Mean Diurnal Range) | 0.178 | 0.327 | 5 |
| Bio_8 (Mean Temp of Wettest Quarter) | 0.162 | 0.332 | 6 |
| T_sand (Topsoil sand content) | 0.155 | 0.33 | 7 |
| T_ece (Topsoil electrical conductivity) | 0.15 | 0.332 | 8 |
| S_silt (Subsoil silt content) | 0.149 | 0.333 | 9 |

**Table S3.** Parameter settings for all component models in the ensemble.

| **Model** | **Key Parameters** | **Notes** |
| --- | --- | --- |
| MaxEnt (optimized) | Regularization multiplier = 2, Feature class = LQH, Max iterations = 1 × 10^3^, Convergence threshold = 1 × 10^−5^ | Optimized via ENMeval (delta AICc = 0) |
| RF | ntree = 500, mtry = sqrt(nvars) | Biomod2 default |
| GBM | n.trees = 1000, interaction.depth = 3, shrinkage = 0.01 | Biomod2 default |
| GAM | Default degrees of freedom (mgcv) | Biomod2 default |
| GLM | Stepwise selection with AIC | Biomod2 default |
| CTA | Complexity parameter (cp) = 0.01 | Biomod2 default |
| ANN | size = 2, decay = 0.01 | Biomod2 default, 5 runs |
| MARS | degree = 2, nk = 10 | Biomod2 default |
| SRE | Not applicable (envelope model) | Biomod2 default |

**Table S4.** Continuous Boyce Index (CBI) for the four models in the final ensemble.

| **Model** | **CBI** |
| --- | --- |
| Random Forest (RF) | 0.992 |
| GAM | 0.973 |
| GBM | 0.956 |
| MaxEnt (optimized) | 0.992 |

**Table S5.** Comparison of AUC values between random cross-validation and spatial block cross-validation for the four models in the final ensemble.

| **Model** | **Spatial CV AUC** | **Spatial CV SD** | **Random CV AUC** | **Random CV SD** |
| --- | --- | --- | --- | --- |
| RF | 0.9639 | 0.0046 | 0.9643 | 0.0132 |
| GAM | 0.9405 | 0.012 | 0.9396 | 0.0154 |
| GBM | 0.9506 | 0.0074 | 0.9519 | 0.0102 |
| MaxEnt (optimized) | 0.9516 | 0.0053 | 0.9531 | 0.0127 |

**Table S6.** Comparison of three ensemble methods for predicting the potential distribution of *C. barometz*.

| **Ensemble method** | **AUC** | **TSS** | **Kappa** |
| --- | --- | --- | --- |
| Equal‑weight averaging | 0.9641 | 0.842 | 0.7944 |
| Weighted averaging (by TSS) | 0.9632 | 0.842 | 0.7944 |
| Consensus voting | 0.8458 | 0.6916 | 0.5913 |

**Table S7.** Suitable area of *C. barometz* under different future climate scenarios and periods.

| **Model** | **Periods** | **Climate scenarios** | **Generally suitable area (×** **104** **km2)** | **Moderately suitable area (** **×** **104** **km2)** | **Highly suitable**  **area (** **×** **104** **km2)** | **Total suitable**  **area (** **×** **104** **km2)** |
| --- | --- | --- | --- | --- | --- | --- |
| MaxEnt | Current | — | 71.08 | 46.74 | 42.94 | 160.76043 |
|  | 2050s | SSP126 | 72.19 | 42.53 | 45.68 | 160.40 |
|  |  | SSP245 | 76.29 | 47.19 | 41.41 | 164.88 |
|  |  | SSP370 | 80.33 | 49.55 | 41.49 | 171.37 |
|  |  | SSP585 | 70.47 | 45.53 | 49.18 | 165.17 |
|  | 2090s | SSP126 | 70.40 | 45.82 | 50.70 | 166.92 |
|  |  | SSP245 | 62.13 | 50.05 | 52.54 | 164.72 |
|  |  | SSP370 | 70.08 | 51.66 | 49.66 | 171.41 |
|  |  | SSP585 | 72.64 | 42.86 | 42.59 | 158.09 |
| RF | Current | — | 77.76 | 45.94 | 39.78 | 163.48 |
|  | 2050s | SSP126 | 61.04 | 43.66 | 40.45 | 145.15 |
|  |  | SSP245 | 101.58 | 0.77 | 0.43 | 102.78 |
|  |  | SSP370 | 109.66 | 0.83 | 0.27 | 110.76 |
|  |  | SSP585 | 110.58 | 0.78 | 0.24 | 111.61 |
|  | 2090s | SSP126 | 79.79 | 57.68 | 0.48 | 137.95 |
|  |  | SSP245 | 73.00 | 64.00 | 1.00 | 138.00 |
|  |  | SSP370 | 110.28 | 0.75 | 0.48 | 111.51 |
|  |  | SSP585 | 110.44 | 0.82 | 0.30 | 111.56 |
| GAM | Current | — | 47.37 | 44.59 | 74.95 | 166.91 |
|  | 2050s | SSP126 | 62.88 | 107.78 | 0.26 | 170.92 |
|  |  | SSP245 | 1.21 | 0.35 | 0.21 | 1.77 |
|  |  | SSP370 | 146.51 | 1.00 | 0.35 | 147.86 |
|  |  | SSP585 | 0.67 | 0.63 | 0.19 | 1.49 |
|  | 2090s | SSP126 | 143.29 | 0.70 | 0.38 | 144.37 |
|  |  | SSP245 | 151.38 | 1.19 | 0.52 | 153.09 |
|  |  | SSP370 | 0.92 | 0.54 | 0.24 | 1.70 |
|  |  | SSP585 | 152.72 | 1.02 | 0.34 | 154.08 |
| GBM | Current | — | 48.43 | 39.26 | 69.64 | 157.33 |
|  | 2050s | SSP126 | 45.88 | 50.36 | 67.83 | 164.07 |
|  |  | SSP245 | 132.82 | 0.87 | 0.45 | 134.14 |
|  |  | SSP370 | 134.84 | 0.97 | 0.33 | 136.14 |
|  |  | SSP585 | 134.76 | 1.02 | 0.25 | 136.03 |
|  | 2090s | SSP126 | 138.88 | 0.75 | 0.41 | 140.04 |
|  |  | SSP245 | 140.86 | 1.08 | 0.52 | 142.46 |
|  |  | SSP370 | 0.82 | 0.66 | 0.25 | 1.74 |
|  |  | SSP585 | 137.40 | 0.97 | 0.33 | 138.70 |

**Table S7 (Continued).** Suitable area of *C. barometz* under different future climate scenarios and periods.

| **Model** | **Periods** | **Climate scenarios** | **Generally suitable area (×** **104** **km2)** | **Moderately suitable area (** **×** **104** **km2)** | **Highly suitable**  **area (** **×** **104** **km2)** | **Total suitable**  **area (** **×** **104** **km2)** |
| --- | --- | --- | --- | --- | --- | --- |
| Ensemble Model | Current | — | 62.25 | 51.12 | 57.30 | 170.67 |
|  | 2050s | SSP126 | 72.18 | 42.60 | 45.69 | 160.47 |
|  |  | SSP245 | 75.36 | 47.70 | 41.89 | 164.95 |
|  |  | SSP370 | 80.31 | 49.57 | 41.49 | 171.37 |
|  |  | SSP585 | 70.46 | 45.54 | 49.20 | 165.21 |
|  | 2090s | SSP126 | 70.38 | 45.85 | 50.73 | 166.96 |
|  |  | SSP245 | 65.77 | 40.20 | 48.52 | 154.49 |
|  |  | SSP370 | 69.85 | 52.50 | 50.13 | 172.48 |
|  |  | SSP585 | 72.68 | 42.94 | 42.38 | 158.00 |

**Table S8** Centroid coordinates and migration distances of suitable habitats for *C. barometz* under different climate scenarios and periods across five modeling approaches (MaxEnt, RF, GAM, GBM, and Ensemble Model).

| **Model** | **Periods** | **Climate scenarios** | **Longitude (° E)** | **Latitude (° N)** | **Migration distance (km)** |
| --- | --- | --- | --- | --- | --- |
|  |  |  |  |  |  |
| MaxEnt | Current | — | 109.02 | 25.89 | — |
|  | 2050s | SSP126 | 109.37 | 25.84 | 35.50 |
|  |  | SSP245 | 108.33 | 25.84 | 68.87 |
|  |  | SSP370 | 108.68 | 25.94 | 35.93 |
|  |  | SSP585 | 109.30 | 25.95 | 28.66 |
|  | 2090s | SSP126 | 110.09 | 25.77 | 72.86 |
|  |  | SSP245 | 104.60 | 25.76 | 367.34 |
|  |  | SSP370 | 105.57 | 25.79 | 288.06 |
|  |  | SSP585 | 107.40 | 25.77 | 190.25 |
| RF | Current | — | 109.15 | 26.24 | — |
|  | 2050s | SSP126 | 108.48 | 26.52 | 73.57 |
|  |  | SSP245 | 105.30 | 31.19 | 666.09 |
|  |  | SSP370 | 106.01 | 32.04 | 713.52 |
|  |  | SSP585 | 105.87 | 31.63 | 678.52 |
|  | 2090s | SSP126 | 108.74 | 28.12 | 179.68 |
|  |  | SSP245 | 107.56 | 28.85 | 339.11 |
|  |  | SSP370 | 104.79 | 30.74 | 185.62 |
|  |  | SSP585 | 104.99 | 31.66 | 84.08 |

**Table S8 (Continued).** Centroid coordinates and migration distances of suitable habitats for *C. barometz* under different climate scenarios and periods across five modeling approaches (MaxEnt, RF, GAM, GBM, and Ensemble Model).

| **Model** | **Periods** | **Climate scenarios** | **Longitude (° E)** | **Latitude (° N)** | **Migration distance (km)** |
| --- | --- | --- | --- | --- | --- |
|  |  |  |  |  |  |
| GAM | Current | — | 107.68 | 27.76 | — |
|  | 2050s | SSP126 | 104.86 | 29.39 | 329.64 |
|  |  | SSP245 | 105.96 | 35.32 | 855.88 |
|  |  | SSP370 | 106.34 | 32.48 | 540.62 |
|  |  | SSP585 | 106.15 | 36.36 | 967.33 |
|  | 2090s | SSP126 | 104.91 | 32.18 | 310.73 |
|  |  | SSP245 | 106.25 | 32.65 | 297.67 |
|  |  | SSP370 | 107.78 | 35.89 | 401.76 |
|  |  | SSP585 | 103.81 | 32.79 | 671.86 |
| GBM | Current | — | 107.73 | 27.15 | — |
|  | 2050s | SSP126 | 106.41 | 27.55 | 138.33 |
|  |  | SSP245 | 105.38 | 32.38 | 624.32 |
|  |  | SSP370 | 107.25 | 32.05 | 547.77 |
|  |  | SSP585 | 106.31 | 32.07 | 564.59 |
|  | 2090s | SSP126 | 106.41 | 31.85 | 477.99 |
|  |  | SSP245 | 107.03 | 32.54 | 155.34 |
|  |  | SSP370 | 107.68 | 35.20 | 352.03 |
|  |  | SSP585 | 105.42 | 32.42 | 92.75 |
| Ensemble Model | Current | — | 108.46 | 26.99 | — |
|  | 2050s | SSP126 | 109.36 | 25.86 | 153.95 |
|  |  | SSP245 | 108.33 | 25.90 | 122.00 |
|  |  | SSP370 | 108.71 | 25.92 | 120.95 |
|  |  | SSP585 | 109.28 | 25.96 | 140.37 |
|  | 2090s | SSP126 | 110.07 | 25.77 | 71.17 |
|  |  | SSP245 | 105.04 | 25.52 | 331.34 |
|  |  | SSP370 | 105.38 | 25.55 | 335.15 |
|  |  | SSP585 | 107.48 | 25.78 | 180.17 |

| **Period** | **Scenario** | **Total suitable area (×10⁴ km²)** | **Centroid longitude (°E)** | **Centroid latitude (°N)** | **Migration distance (km)** |
| --- | --- | --- | --- | --- | --- |
| Current | — | 156.44 | 109.834 | 25.682 | — |
| 2050s | SSP126 | 157.82 | 110.507 | 26.096 | 81.51 |
| 2050s | SSP245 | 190.66 | 107.777 | 26.334 | 218.03 |
| 2050s | SSP370 | 178.66 | 109.169 | 26.752 | 136.02 |
| 2050s | SSP585 | 173.02 | 109.008 | 26.256 | 104.34 |
| 2090s | SSP126 | 178.22 | 110.353 | 26.069 | 15.62 |
| 2090s | SSP245 | 188.36 | 105.43 | 25.464 | 253.87 |
| 2090s | SSP370 | 170.9 | 107.466 | 25.698 | 206.23 |
| 2090s | SSP585 | 166.41 | 108.41 | 26.14 | 61.02 |

**Table S9.** Suitable area and centroid shifts for *C. barometz* based on 128 temporally filtered occurrence points (1995–2025) under different climate scenarios and periods (MaxEnt model). Model performance: mean test AUC = 0.953 (SD 0.006).

**Table S10.** Suitable area and centroid shifts for *C. barometz* based on the fixed threshold (10th percentile training presence Cloglog = 0.3927) under different climate scenarios and periods (MaxEnt model).

| **Period** | **Scenario** | **Suitable area**  **(×10⁴ km²)** | **Centroid longitude (°E)** | **Centroid latitude (°N)** | **Migration distance (km)** |
| --- | --- | --- | --- | --- | --- |
| Current | — | 76.75 | 107.78 | 26.87 | — |
| 2050s | SSP126 | 77.95 | 107.29 | 25.94 | 115.15 |
| 2050s | SSP245 | 83.68 | 107.01 | 26.37 | 95.1 |
| 2050s | SSP370 | 81.59 | 107.69 | 26.38 | 55.77 |
| 2050s | SSP585 | 82.8 | 107.69 | 25.75 | 125.16 |
| 2090s | SSP126 | 80.45 | 108.2 | 26.04 | 92.03 |
| 2090s | SSP245 | 82.46 | 104.98 | 24.38 | 300.53 |
| 2090s | SSP370 | 79.99 | 106.81 | 24.91 | 185.04 |
| 2090s | SSP585 | 80.84 | 106.03 | 25.52 | 168.01 |

Note: The fixed threshold (0.3927) was derived as the mean 10th percentile training presence Cloglog threshold from 10‑fold cross‑validation of the optimized MaxEnt model under current climate. Compared with the Natural Breaks method, the direction of centroid shift was fully consistent across all scenarios (southwestward under SSP245, SSP370, SSP585; southeastward under SSP126), confirming that the core conclusion of this study is robust to the choice of classification method.
